# Incorporation of a metal-mediated base pair into an ATP aptamer – using silver(I) ions to modulate aptamer function

**DOI:** 10.3762/bjoc.16.236

**Published:** 2020-11-25

**Authors:** Marius H Heddinga, Jens Müller

**Affiliations:** 1 Institut für Anorganische und Analytische Chemie & Cells in Motion Interfaculty Center, Westfälische Wilhelms-Universität Münster, Corrensstr. 28/30, 48149 Münster, Germany

**Keywords:** aptamer, ATP, bioinorganic chemistry, DNA, imidazole, metal-mediated base pairs

## Abstract

For the first time, a metal-mediated base pair has been used to modulate the affinity of an aptamer towards its target. In particular, two artificial imidazole 2’-deoxyribonucleosides (Im) were incorporated into various positions of an established ATP-binding aptamer (ATP, adenosine triphosphate), resulting in the formation of three aptamer derivatives bearing Im:Im mispairs with a reduced ATP affinity. A fluorescence spectroscopy assay and a binding assay with immobilized ATP were used to evaluate the aptamer derivatives. Upon the addition of one Ag(I) ion per mispair, stabilizing Im–Ag(I)–Im base pairs were formed. As a result, the affinity of the aptamer derivative towards ATP is restored again. The silver(I)-mediated base-pair formation was particularly suitable to modulate the aptamer function when the Im:Im mispairs (and hence the resulting metal-mediated base pairs) were located close to the ATP-binding pocket of the aptamer. Being able to trigger the aptamer function opens new possibilities for applications of oligonucleotides.

## Introduction

Aptamers are oligonucleotides capable of recognizing and binding to specific molecules up to the size of proteins [1]. While mostly unstructured in solution, aptamers typically fold into their three-dimensional structure upon binding the respective target molecule [2]. Aptamers are normally identified by the in vitro selection from combinatorial libraries of nucleic acids [3]. In the past decades, interest in aptamers has risen significantly, as they can be applied in a variety of disciplines, e.g., as therapeutics or as sensors [4–7].

Another topical area of research in the field of nucleic acids involves the development of nucleic acids whose structure (and therefore function) can be regulated by the addition (or removal) of external triggers, allowing to switch the nucleic acid function [8]. For example, DNA can be used in nanotechnology to create mechanically moving systems such as walkers, fueled by the addition of appropriately designed oligonucleotides [9]. Moreover, external triggers can be applied to release a DNA-bound cargo (in the form of small organic molecules) [10]. Similarly, metal ions can trigger DNA folding into a catalytically active topology [11–12].

Metal-mediated base pairs are artificial base pairs in which hydrogen bonds between the complementary nucleobases are formally replaced by coordinate bonds to one (or more) transition metal ions [13–14]. They are compatible with normal DNA duplex structures [15–16] and can be used to introduce a metal-based functionality into DNA [17–18]. Importantly, metal-mediated base-pair formation has been successfully applied in metal-responsive structural transformations of various types [19–21]. We therefore envisaged the development of an aptamer whose function would be modulated by the formation of a metal-mediated base pair. So far, only one example exists of such a rationally designed aptamer. In that work, a thrombin-binding aptamer that adopts a guanine quadruplex structure was modified by four pyridine ligands [22]. The addition of Cu(II) or Ni(II) ions leads to the formation of a square-planar complex that reduces the affinity of the modified aptamer to its target protein. To the best of our knowledge, there is no precedence for an aptamer whose affinity is restored upon metal-mediated base-pair formation. One example exists of a Ag(I)-binding aptamer, but that aptamer does not rely on the formation of a Ag(I)-mediated base pair [23–24].

The aptamer selected for this study is the ATP-binding aptamer (ATP, adenosine triphosphate) [25]. The choice of this aptamer was expected to have various advantages. 1) Its three-dimensional structure has been reported [26], so the position for the incorporation of the metal-mediated base pair can be chosen in a meaningful way. 2) Its structure is based on a duplex rather than a quadruplex, so that metal-mediated base pairs can be introduced. 3) It is a short oligonucleotide, so the modified aptamers can easily be synthesized using automated solid-phase synthesis. 4) The target molecule of this aptamer is a small molecule, facilitating the binding assays. 5) The aptamer also binds AMP (adenosine monophosphate) with high affinity [25], which is less prone to hydrolysis than ATP and can therefore comfortably be used in the binding assays. 6) Finally, the ATP aptamer has already been exploited in many other contexts such as in DNA origami [27–29], indicating its high versatility.

**Figure 1:**
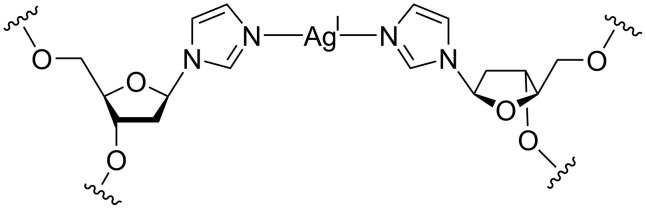
Chemical structure of the Im–Ag(I)–Im base pair [30].

The metal-mediated base pair chosen for this study is based on the artificial imidazole 2’-deoxyribonucleoside (Im). This nucleoside is well known to form highly stabilizing Ag(I)-mediated Im–Ag(I)–Im base pairs (Figure 1) [30–33] and has already been proposed for a use in various applications [34–36]. A slight disadvantage of using this metal-mediated base pair could be the fact that Ag(I) is known to have an affinity to adenine derivatives (such as ATP, i.e., the target molecule of the aptamer) [37–38]. However, as the incorporation into an Im–Ag(I)–Im base pair should be favoured by the chelate effect, compared to the binding to ATP, the combination of ATP-binding aptamer and Im–Ag(I)–Im base pair was finally selected for this study.

## Results and Discussion

**Figure 2:**
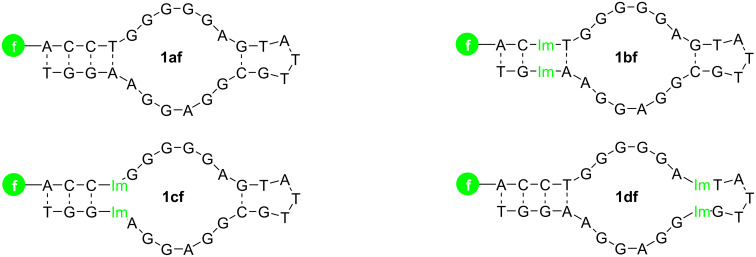
The aptamer derivatives used in this study. The imidazole deoxyribonucleotide is marked in green as Im, while f represents fluorescein.

Four ATP/AMP aptamer derivatives were synthesized and terminally labeled with a fluorescein moiety. Three of these have Im:Im pairs inserted at different positions into the sequence (Figure 2). The oligonucleotide **1af** is essentially identical to the established ATP/AMP-binding aptamer, with the attached fluorescein moiety representing the sole difference. The oligonucleotide **1bf** bears one Im:Im pair located two base pairs away from the 5’ and 3’ ends of the aptamer. The aptamer derivatives **1cf** and **1df** contain one Im:Im pair directly adjacent to the central binding pocket. For **1cf**, the Im:Im pair is located towards the termini, whereas in derivative **1df** it is close to the loop region.

### Melting curves and CD spectroscopy

To evaluate whether the Ag(I)-mediated base pairs are indeed formed in the aptamer derivatives, melting curves and circular dichroism (CD) spectra were recorded in the presence of increasing amounts of Ag(I). The DNA melting is expected to occur when the loop structure of the aptamers opens up.

**Figure 3:**
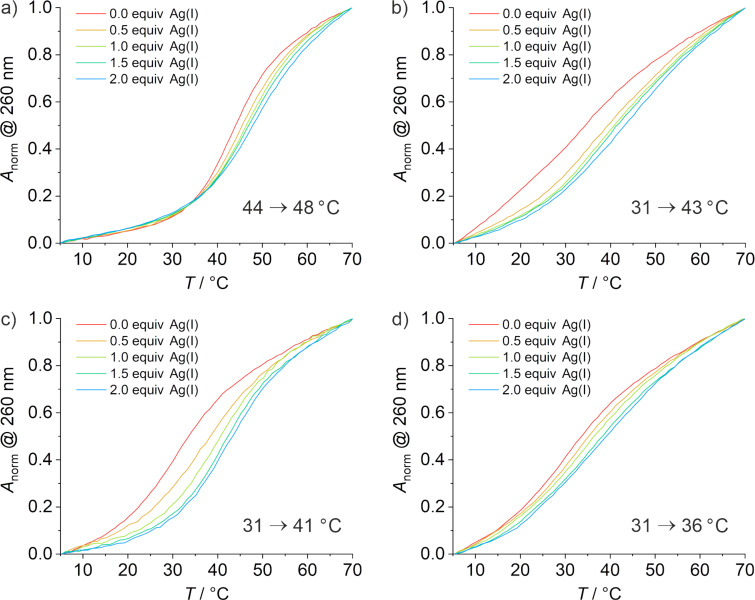
Temperature-dependent normalized UV absorbance at 260 nm for oligonucleotides a) **1af**, b) **1bf**, c) **1cf**, and d) **1df** in the absence (red line) and presence of increasing amounts of Ag(I). The melting temperatures in the presence of 0.0 and 1.0 equiv of Ag(I) are given, too. Conditions: 1 μM oligonucleotide, 50 mM NaClO_4_, 5 mM MOPS buffer (pH 6.8).

In the absence of Ag(I), aptamer **1af** shows the highest melting temperature *T*_m_ of all aptamer derivatives (44 °C, Figure 3). In contrast, the modified aptamers melt at a significantly lower temperature (31 °C). As these derivatives bear the Im:Im pair directly adjacent to the ATP binding pocket, it can be concluded that the nucleobases close to the binding pocket contribute to a large extent to the aptamer stability. All imidazole-containing aptamer derivatives (**1bf**, **1cf**, **1df**) show a significant increase in *T*_m_ upon the addition of the first equivalent of Ag(I) but only minor additional changes in the presence of excess Ag(I). This is a typical indication for the formation of a Ag(I)-mediated base pair [17]. It is therefore likely that the increased stability is caused primarily by the formation of one Im–Ag(I)–Im base pair. In contrast, the aptamer **1af** lacking any imidazole residues shows a much smaller increase in *T*_m_. Moreover, the *T*_m_ of **1af** increases more or less steadily with an increasing Ag(I) concentration, suggesting non-specific interactions between Ag(I) and the canonical nucleobases in **1af**.

To obtain further insight into the Ag(I)-binding behavior of the aptamer derivatives, CD spectra were recorded (Figure S1 in Supporting Information File 1). All aptamer derivatives show a positive Cotton effect between 260 and 290 nm as well as a negative Cotton effect between 220 and 260 nm, indicating a B-type DNA structure [39]. For the most part, significant changes or trends cannot be observed in the CD spectra, indicating that the formation of the Ag(I)-mediated base pairs as deduced from the melting curves does not influence the overall DNA topology. This is a well-known feature of the Im–Ag(I)–Im base pair [31].

### Fluorescence spectroscopy assay

**Figure 4:**
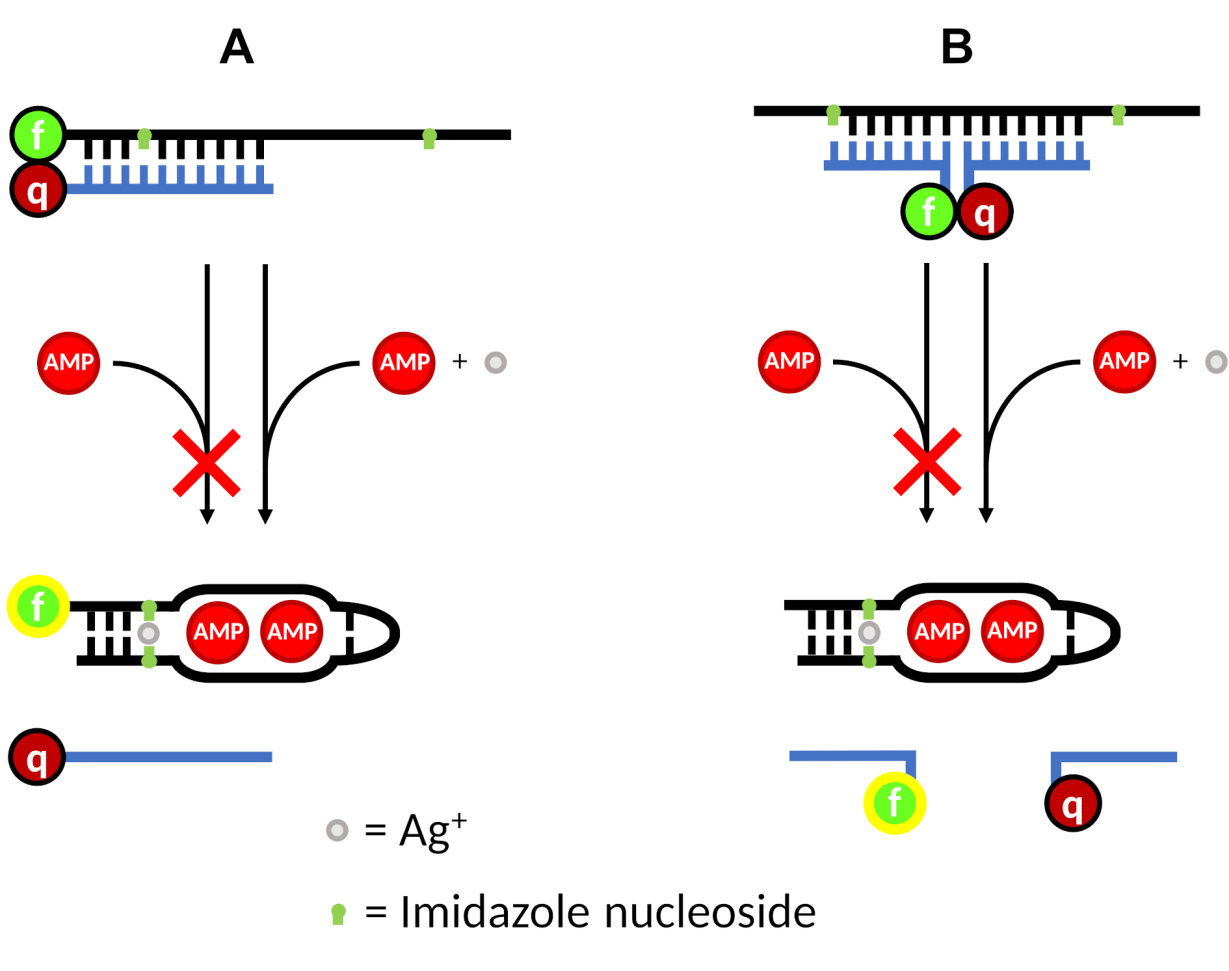
The two systems devised to investigate the envisaged functionality of the modified structure-switching signaling aptamers (f = fluorescein, q = DABCYL). The strand shown in black is derived from the ATP/AMP-binding aptamer.

**Figure 5:**
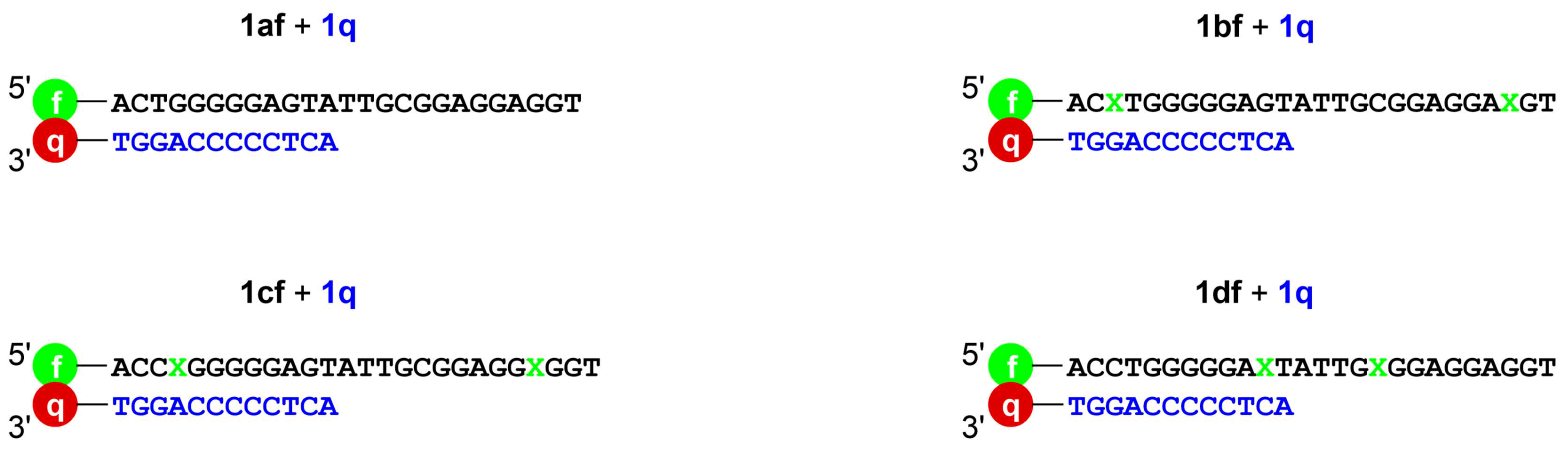
System A: the aptamers **1af**, **1bf**, **1cf**, and **1df** with the complementary sequence **1q** (X = imidazole, f = fluorescein, q = DABCYL).

In 2003, the ATP/AMP-binding aptamer was applied in combination with a fluorophore (fluorescein), a quencher (DABCYL), and complementary oligonucleotides to devise a system referred to as structure-switching signaling aptamer [40]. Based on this concept, two systems were devised to investigate whether the Im–Ag(I)–Im base pairs could be applied to manipulate the formation of the aptamer:AMP complex. System A utilizes the 5’-fluorescein-labeled imidazole-containing oligonucleotide derived from the aptamer (**1af**, **1bf**, **1cf**, **1df**) in combination with a complementary oligonucleotide (**1q**, quencher-labeled DNA) bearing a DABCYL moiety at the 3’ terminus (Figure 4A and Figure 5). In the absence of AMP, the sequences should form a regular duplex structure, with the fluorescence of the fluorescein being quenched by the DABCYL moiety located nearby. In the presence of AMP and Ag(I), the aptamer should instead form the aptamer:AMP complex, releasing the quencher-labeled DNA and henceforth restoring the fluorescence. Ideally, if the aptamer is suitably modified with imidazole (Figure 4A), the aptamer:AMP complex should be too unstable to form in the absence of Ag(I). In this case, the system should only fluoresce when both AMP and Ag(I) are present. System B functions in a similar manner (Figure 4B), but instead of the aptamer carrying the fluorescent label, it utilizes two complementary sequences, one carrying fluorescein (**2f**) and the other carrying DABCYL (**2q**).

**Figure 6:**
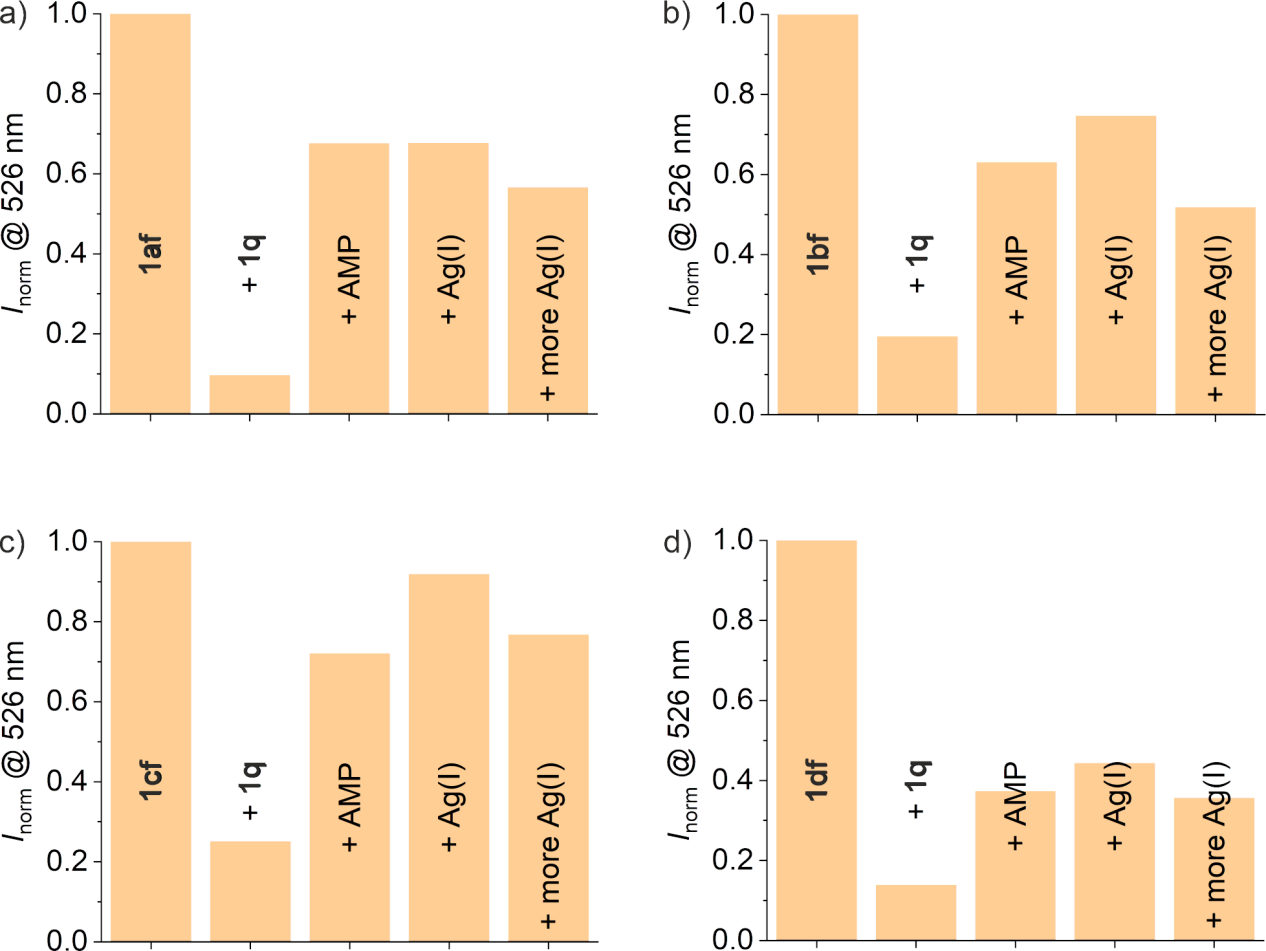
Normalized fluorescence intensity at 526 nm (λ_exc_ = 490 nm) of a) **1af**, b) **1bf**, c) **1cf**, and d) **1df** prior to and after the successive addition of **1q**, 1 mM AMP, 1 equiv of Ag(I), and 9 more equiv of Ag(I), respectively. After each addition and before the respective measurement, the solution was heated to 40 °C for 10 min and then kept at 22 °C for 30 min (conditions: 40 nM fluorophore-labeled DNA, 80 nM **1q**, 300 mM NaClO_4_, 5 mM Mg(ClO_4_)_2_, and 20 mM MOPS buffer (pH 7.4)).

First, the change in fluorescence was investigated for all sequences of the system A via fluorescence spectroscopy. The measurements were conducted at pH 7.4 and 8.1. In both cases, similar results were obtained. Hence, the data for pH 7.4 will be presented and discussed in the following (Figure 6), whereas the data for pH 8.1 can be found in Supporting Information File 1 (Figure S2). The fluorescence intensities were normalized according to *I*_norm_* = F/F*_0_, wherein *F* = fluorescence intensity and *F*_0_ = fluorescence intensity of the fluorescein-labeled oligonucleotide without any additions. As expected, the oligonucleotide **1af** shows the most efficient quenching upon the addition of **1q** with a 90% decrease in emission. Upon the addition of AMP, the emission increases again almost sevenfold, indicating a dissociation of the quencher-labeled oligonucleotide and subsequent formation of the AMP-bound structure. Adding 1 equiv of Ag(I) leads to no significant further change, as was expected for the imidazole-free derivative **1af**. Nevertheless, the addition of 10 equiv of Ag(I) leads to a slight decrease in the emission. The oligonucleotides **1bf**, **1cf**, and **1df** all show less efficient quenching upon the addition of **1q**, which can be attributed to the presence of the destabilizing mismatches involving imidazole. This effect is least pronounced for oligonucleotide **1df**, which is quenched almost as effectively as **1af**. All imidazole-containing aptamer derivatives show an increased emission upon the addition of AMP with an approximately threefold increase in the intensity. In contrast to aptamer **1af**, however, they show a slight additional increase in emission upon the addition of 1 equiv of Ag(I). This effect is most pronounced for the derivative **1cf**. Like **1af**, the imidazole-containing aptamer derivatives show a decrease in intensity in the presence of excess Ag(I). This common observation implies that the first slight increase upon the addition of 1 equiv of Ag(I) may be attributed to the formation of an Im–Ag(I)–Im base pair. The decreased emission with excess of Ag(I) may then be caused by less specific interactions between the Ag(I) ions and DNA, as Ag(I) is known to also bind to natural nucleobases [41–43]. Again, this non-specific binding of excess Ag(I) can be deduced from the melting curves, as is exemplarily shown for the duplex formed from **1af** and **1q** (Figure S3a in Supporting Information File 1). It is also notable that of all aptamer derivatives, **1df** shows the lowest fluorescence intensity in the presence of AMP, indicating an overall reduced affinity of **1df** to AMP.

It was further investigated whether the order of the addition of AMP and Ag(I) influences the outcome of the experiment, as it may be anticipated that free AMP competes for the binding of Ag(I). However, no significant changes in the level of emission after the addition of both Ag(I) and AMP were observed (see Supporting Information File 1, Figure S4).

A variant of system A, in which the quencher oligonucleotide **1q** is replaced by the slightly longer sequence **1q2**, was investigated as well. The two additional bases of **1q2** should be able to form two more canonical base pairs with the aptamer and therefore compensate for the destabilization caused by the imidazole mismatches. As expected, all fluorophore-labeled aptamers show a more effective quenching upon the addition of **1q2** (Figure S5 in Supporting Information File 1). The relative changes in the emission are quite similar to the ones observed with **1q** though.

Again, the measurements were repeated with Ag(I) being added prior to AMP. While adding 1 equiv of Ag(I) leads to only marginal changes in the emission, adding Ag(I) in excess leads to a significant increase in fluorescence intensity for all sequences (Figure S6 in Supporting Information File 1). As this change is not limited to the imidazole-containing aptamer derivatives, it is probably caused by non-specific interactions of the Ag(I) ions with the natural nucleobases. Interestingly, when the shorter quencher sequence **1q** is used, excess Ag(I) leads to a decrease in the emission when AMP is present already. As to why **1q2** behaves differently is unclear. Furthermore, while the relative increase in the emission caused by Ag(I) is similar for all sequences, **1cf** shows a much greater change when AMP is added. Possibly, **1cf** possesses a higher affinity to AMP under these conditions than the other sequences.

**Figure 7:**
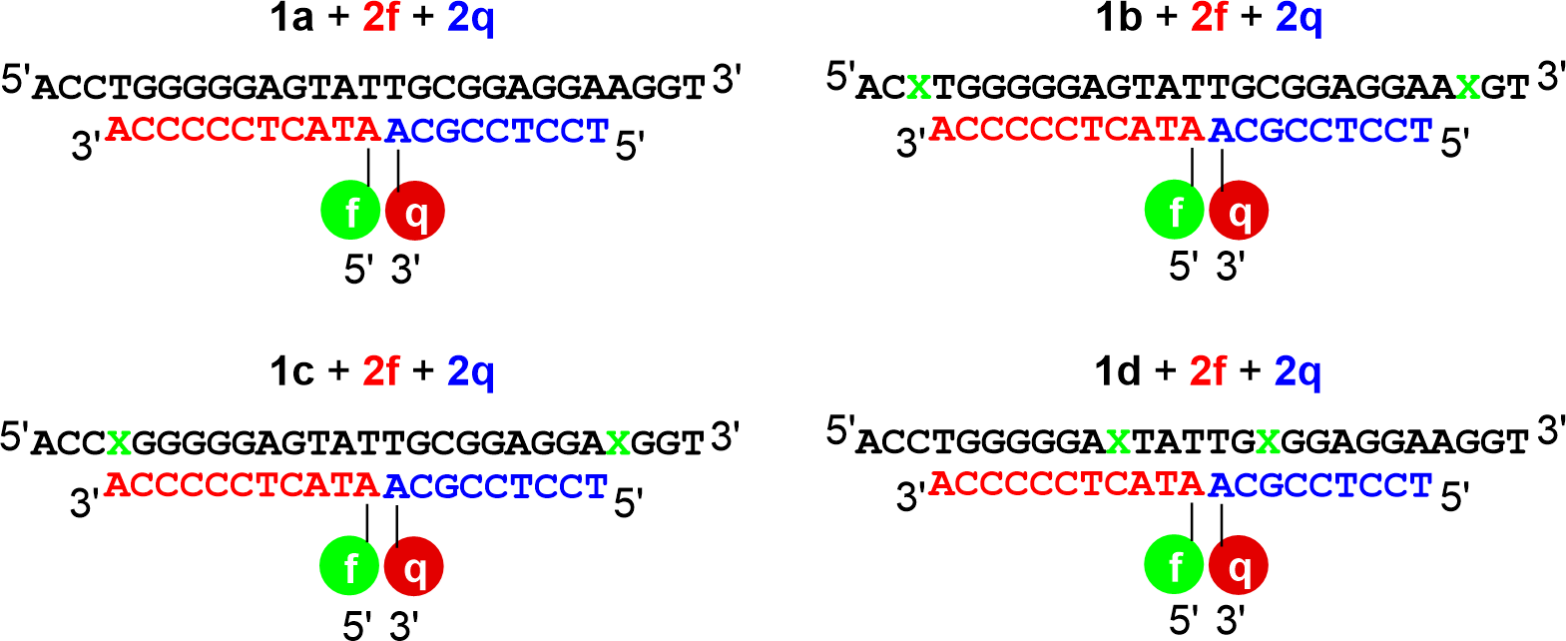
System B: aptamers **1a**, **1b**, **1c**, and **1d** with the complementary oligonucleotides **2f** and **2q** (X = imidazole, f = fluorescein, q = DABCYL).

In a second set of fluorescence experiments, system B was investigated analogously. In the system B, the fluorescein moiety is not attached to the aptamer derivative but to the oligonucleotide **2f** (Figure 7).

**Figure 8:**
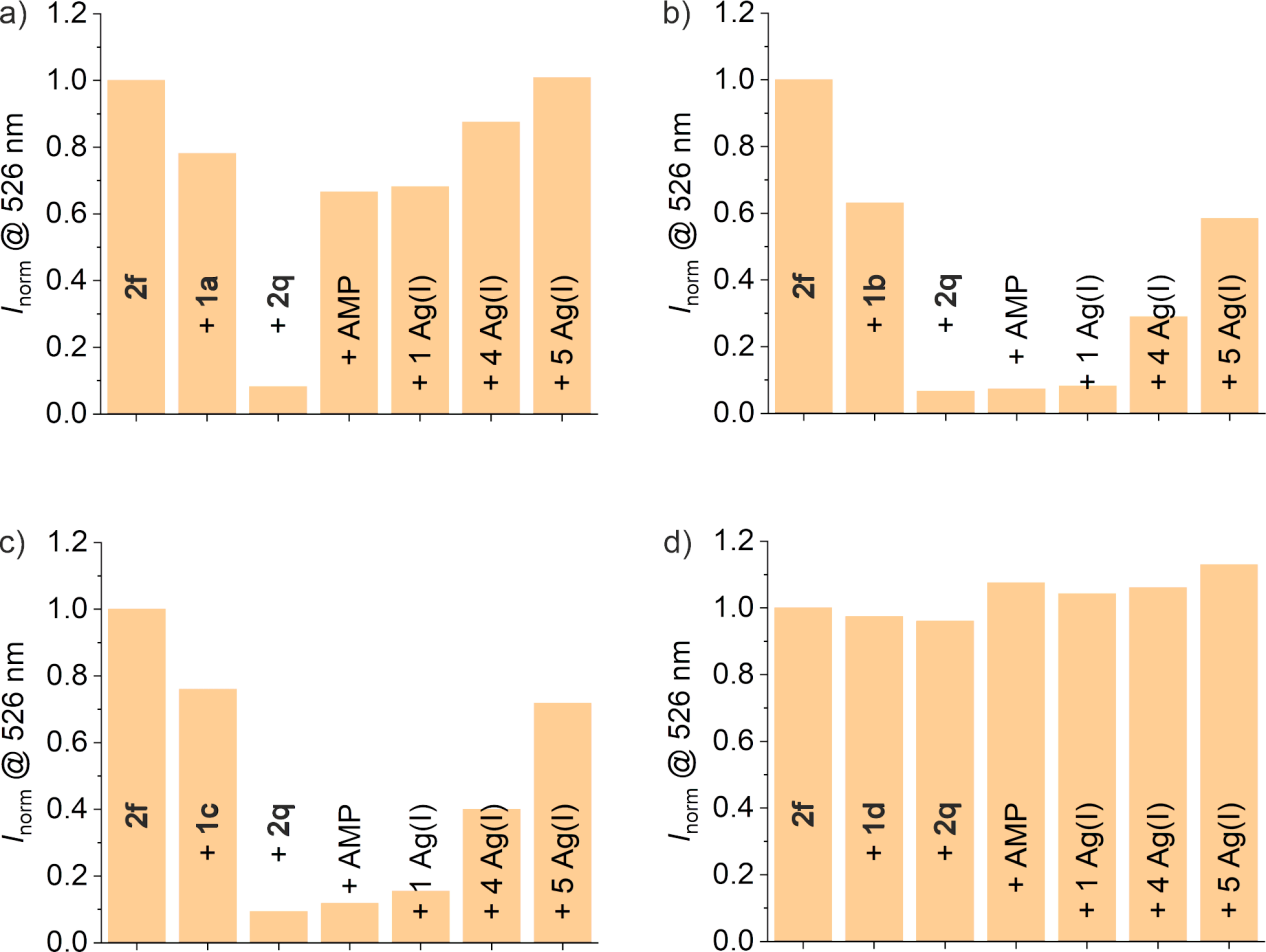
Normalized fluorescence intensity at 526 nm (λ_exc_ = 490 nm) of the oligonucleotide **2f** before and after the successive addition of the aptamer (derivatives) a) **1a**, b) **1b**, c) **1c**, and d) **1d**, of **2q**, of 1 mM AMP, and of increasing equiv of Ag(I). After every addition and before the respective measurement, the solution was heated to 40 °C for 10 min and then kept at 22 °C for 30 min (conditions: 40 nM **2f**; 80 nM aptamer derivative; 120 nM **2q**, 300 mM NaClO_4_, 5 mM Mg(ClO_4_)_2_, and 20 mM MOPS buffer (pH 8.1)).

The fluorescence of **2f** is defined as 100% in this set of experiments, as it was found that the presence of the aptamer derivatives significantly reduces the fluorescence. It should be reasonable to assume that the degree to which the emission is quenched upon the aptamer addition serves as a first indicator of the stability of the resulting duplex. As such, the addition of oligonucleotides **1a** and **1b**, which do not form mismatched base pairs with the oligonucleotide **2f**, as well as **1c**, which only forms one mismatched pair at the 3’ terminus of **2f**, results in a similar reduction in the fluorescence (Figure 8). The addition of oligonucleotide **1d**, which forms one mismatched base pair with **2f** close to the fluorescein moiety, leads to hardly any reduction in the fluorescence emission, indicating that **2f** binds less effectively (if at all). The oligonucleotide **2q** quenches the fluorescence of the duplexes formed by **2f** and **1a**, **1b** or **1c** with similar effectiveness, while almost no quenching occurs with **1d**. This can be attributed to either **2q** or **2f** (or both) not binding effectively to **1d**, which would be in line with the fact that in both cases one imidazole-containing mismatch would be present in the duplex. Except for the system involving the aptamer **1a**, none of the systems shows a significant response to AMP in the absence of Ag(I). Unexpectedly, even after the addition of 1 equiv of Ag(I), the fluorescence emission does not significantly increase either. Adding an excess of Ag(I) does increase the emission in all cases. This effect hence could be caused by unspecific interactions between Ag(I) and the canonical nucleobases and cannot be attributed to the formation of Im–Ag(I)–Im base pairs. Interestingly, such proposed unspecific interactions do not lead to an increase in *T*_m_ for the duplexes formed from **1a**, **2f** and **2q** (Figure S3b, Supporting Information File 1), which could possibly be due to the ternary nature of this duplex. It appears as if the presence of excess Ag(I) somehow disrupts the binding of **2f** and **2q** to the aptamer derivatives.

### Assay with immobilized ATP

To finally evaluate whether the incorporation of Im:Im pairs into the aptamer allows for its target affinity to be manipulated, a binding assay using ATP-agarose gel was performed [44]. Towards this end, the aptamer derivatives **1af**–**1df** were loaded onto an agarose gel containing immobilized ATP, followed by washing with buffer. The elution was quantified by UV–vis spectroscopy.

**Figure 9:**
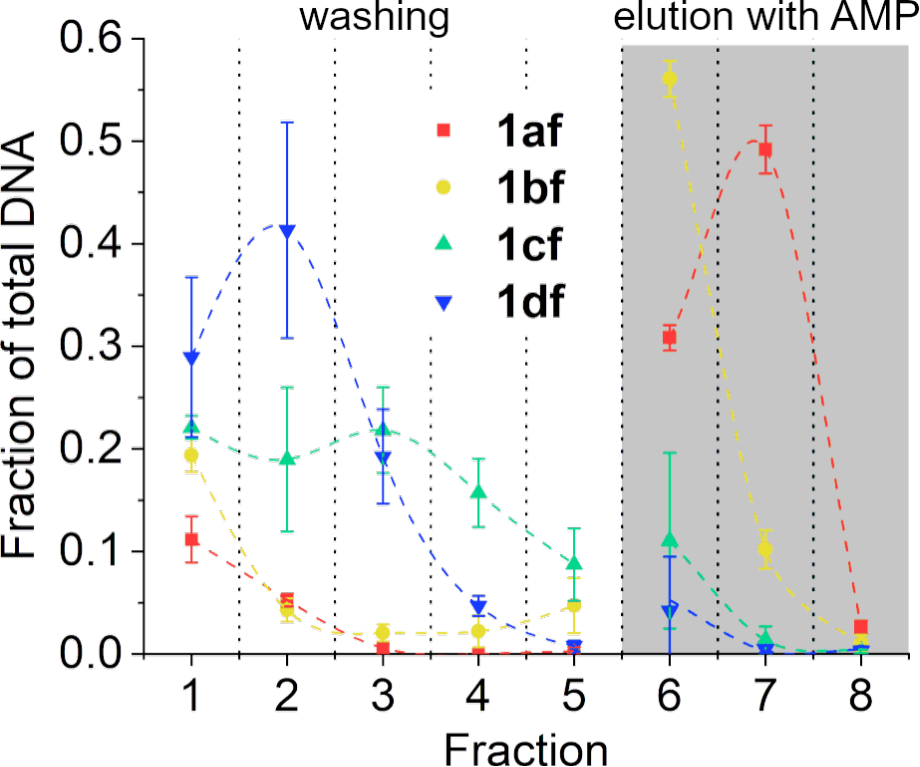
Elution of the aptamer derivatives from the ATP-agarose gel in the absence of Ag(I): **1af** (red), **1bf** (yellow), **1cf** (green), and **1df** (blue) (*n* = 3). Conditions: 1 μM oligonucleotide, 300 mM NaClO_4_, 5 mM Mg(ClO_4_)_2_, and 20 mM MOPS buffer (pH 7.4).

In the absence of Ag(I), the aptamer derivatives bearing imidazole residues show a lower affinity to ATP than the unmodified aptamer (Figure 9). While only 17% of the DNA is eluted in the five washing fractions of **1af**, the amount of eluted DNA is almost twice as high for **1bf** with 32% being washed off the column. This effect is even more pronounced for the derivatives **1cf** and **1df**, which are almost completely eluted in the five washing fractions (87% and 95%, respectively). As seen before in the fluorescence spectroscopy assay (vide supra), the presence of an Im:Im mismatch directly adjacent to the binding pocket of the aptamer significantly disrupts its ability to bind ATP. Locating the mismatch further away from the binding pocket has a much less pronounced impact. Particularly the G:C pair close to the loop region that has been replaced by an Im:Im mismatch in **1df** seems to be important for the stability of the aptamer:ATP complex. As can be seen in the elution profiles, **1df** is eluted fastest from the column (Figure 9).

**Figure 10:**
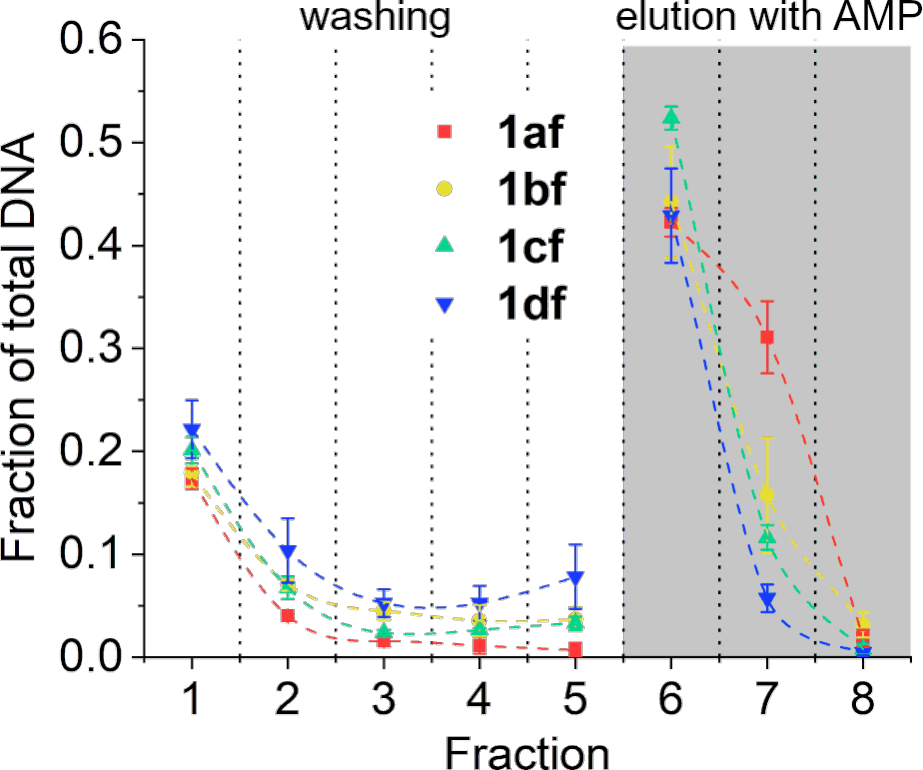
Elution of the aptamers from the ATP-agarose gel in the presence of 1 equiv of Ag(I): **1af** (red), **1bf** (yellow), **1cf** (green), and **1df** (blue) (*n* = 3). Conditions: 1 μM oligonucleotide, 300 mM NaClO_4_, 5 mM Mg(ClO_4_)_2_, and 20 mM MOPS buffer (pH 7.4).

Adding 1 equiv of Ag(I) to the aptamer solution prior to conducting the binding assay leads to significant changes in the affinity particularly of **1cf** and **1df** to the immobilized ATP (Figure 10). With 35% of the aptamer being eluted during the five washing steps, **1cf** now shows essentially the same ATP affinity as **1bf**. The affinity of **1df** remains the lowest of all aptamer derivatives. However, even with this derivative an almost tenfold increase is found. It is noteworthy that the elution profiles of the modified aptamers after the addition of Ag(I) are very similar to the one of **1af** (Figure 10). The increased affinity of the derivatives can likely be attributed to the formation of stable Im–Ag(I)–Im base pairs, as the unmodified aptamer does not show an increase in affinity upon the addition of Ag(I). The position of the metal-mediated base pair seems to be of great importance, as the most significant increases in affinity are observed when the Im–Ag(I)–Im base pair is situated directly adjacent to the ATP binding pocket. When located in-between the Watson–Crick base pairs of the stem region, as in the case of **1bf**, no significant increase in the affinity is observed.

To evaluate whether the aptamers are still specific when modified with the imidazole moiety, their affinity for GMP (guanosine monophosphate) was probed, too. As expected, the unmodified aptamer **1af** cannot be eluted from the ATP affinity column by means of a GMP solution (see Supporting Information File 1, Figure S7a). Similarly, the aptamer derivatives **1bf**, **1cf**, and **1df** are not eluted by GMP either (see Supporting Information File 1, Figure S7b). This strongly suggests that the modified aptamers are still specific.

The binding assays were repeated using the respective aptamers lacking the fluorescein label (**1a**, **1b**, **1c**, and **1d**; Figure S8 in Supporting Information File 1). Without the fluorescent marker, a quantification of the eluted oligonucleotide has to be performed by using UV spectroscopy. Hence, only the amount of DNA eluted during the initial five washing steps can be quantified. It is impossible to determine the amount of the oligonucleotide eluted by the AMP solution, as AMP absorbs in the same wavelength range as DNA. It is, however, possible to compare the elution profiles during the washing phase. Interestingly, the data for aptamer **1b** differ somewhat from those of the fluorescein-labeled oligonucleotide **1bf**. For example, in the absence of Ag(I), **1b** shows a far lower ATP affinity than **1bf** with almost the entire DNA being eluted during the washing steps. On the other hand, the aptamers **1c** and **1d** show comparable elution profiles, as do the derivatives **1cf** and **1df**. In the presence of 1 equiv of Ag(I), the assays indicate a restored affinity towards ATP for **1c** and **1d**, similar to that of aptamer **1a** without imidazole moieties. Surprisingly, the affinity of **1b** remains almost unchanged.

Overall, it seems that the fluorescein label influences the affinity of the aptamers to ATP only in the case of **1b**/**1bf** where the imidazole moiety is located closest to the fluorophore.

## Conclusion

The metal-mediated Im–Ag(I)–Im base pair can be used to modulate the affinity of the ATP-binding aptamer to its target. Placing an Im:Im mispair close to the ATP binding pocket significantly reduces the aptamer affinity, whereas a localization closer to the end of the duplex, where fraying is possible, has less of an influence. Importantly, the addition of Ag(I), which is incorporated into the Im:Im mispair to form a stabilizing Im–Ag(I)–Im pair, largely restores the ability of the aptamer derivative to bind to its target. The ability to modulate the aptamer affinity by adding suitable transition metal ions extends the applicability of aptamers even further, as it allows triggering the function at any desired point. Further optimizations will be necessary to eliminate the effect of excess transition metal ions and to enable a more complete switching of the aptamer function. This could be possible by using other metal-mediated base pairs, based either on other metal ions or on other artificial nucleobases.

## Experimental

The reagents were purchased from Acros Organics, ABCR, Sigma-Aldrich, Activate Scientific, and Alfa Aesar and were used without further purification. The solvents used were purchased from VWR, Sigma-Aldrich, ABCR, Fluka, Fisher Scientific, and Merck. Dry pyridine was purchased from Acros Organics*.* Dichloromethane was distilled and dried over molecular sieves. Milli-Q water was used to prepare solutions and buffers. The silica gel used for product purification (Geduran^®^ Si60, 40–63 µm) was purchased from Merck.

The DMT- and phosphoramidite-protected imidazole nucleoside as required for automated DNA solid-phase synthesis was synthesized as previously reported [30]. The oligonucleotides were synthesized on a DNA/RNA synthesizer H-8 (K&A Laborgeräte) using standard protocols for automated solid-phase synthesis (coupling time: 1000 s for unnatural nucleosides). All natural nucleosides (A, T, G, and C), the fluorescein phosphoramidite as well as the DABCYL-CPGs were obtained from Glen Research. The oligonucleotides were deprotected and cleaved from the support by incubation in AMA solution (1:1, 40% methylamine and aqueous ammonia) at 65 °C for 15 min. They were purified using denaturing polyacrylamide gel electrophoresis (gel solution: 7 M urea, 1 TBE buffer, 14 or 18% (depending on the length of the oligonucleotide) polyacrylamide/bisacrylamide (29:1); loading buffer: 11.8 M urea, 42 mM Tris·HCl (pH 7.5), 0.83 mM EDTA (pH 8.0), 8% sucrose), and desalted using NAP 10 columns. MALDI–TOF spectrometry was used to characterize the oligonucleotides either in a 3-hydroxypicolinic acid (3-HPA)/ammonium acetate matrix or in a 3-HPA in TA50 solvent matrix (50:50, v/v acetonitrile/0.1% TFA in water) containing 10 mg/mL diammonium hydrogen citrate (Supporting Information File 1, Table S1, Figures S9–S20). The purity was confirmed by means of HPLC chromatography (Figures S21–S32, Supporting Information File 1). Towards this end, a Macherey–Nagel NUCLEODUR C18 HTec column (5 μm, 124 × 4 mm) was used in combination with the following eluents at a flow rate of 1 mL/min: trimethylamine/acetic acid 1:1, 10 mM in water (solvent A) and trimethylamine/acetic acid 1:1, 10 mM in water/CH_3_CN 1:4 (solvent B). Gradient applied: 0–5 min, 3% B; 5–45 min, 3–40% B; 45–50 min, 100% B. The quantification of the sequences was achieved using a Thermo Scientific NanoDrop 2000c photospectrometer. The molar extinction coefficients of the oligonucleotides were calculated from the known ε_260_ values of the natural nucleobases as well as fluorescein and DABCYL. An ε_260_ of 0.0 was used for the imidazole deoxyribonucleoside.

The binding assays were conducted using an ATP-loaded agarose gel obtained from Sigma-Aldrich (cross-linked 4% beaded agarose, ATP is attached at its C8 position via a 9C linker, 1–5 μmol ATP per mL gel). For the binding assay, 5 nmol DNA in 200 μL column buffer (300 mM NaClO_4_, 5 mM Mg(ClO_4_)_2_, 20 mM MOPS (pH 7.4)) were loaded onto 500 μL of agarose gel pre-equilibrated in column buffer. After 5 min, the column was washed five times with column buffer (1 mL) and then eluted three times with 3 mM AMP (or GMP) solution in column buffer (750 μL each). Each fraction was collected individually, lyophilized, and the residue dissolved in 1 mL water. The DNA was quantified using the NanoDrop 2000c and the calculated ε_260_ values of the oligonucleotides as well as the ε_490_ value of fluorescein. When conducting the binding assays in the presence of Ag(I), 1 equivalent of AgClO_4_ was added to 5 nmol DNA in 200 μL column buffer and the solution was left at room temperature for 15 min before applying it to the column. The assay was performed in triplicate.

Fluorescence spectra were recorded on a Chirascan V100 with CCD fluorometer instrument with excitation at 490 nm, an emission range of 200–995 nm, and a data interval of 0.8 nm. First, the fluorescence intensity of the fluorescein-labeled DNA was measured at 40 nM in buffer (300 mM NaClO_4_, 5 mM Mg(ClO_4_)_2_, 20 mM MOPS (pH 8.1)) at 22 °C. After adding the quencher-labeled DNA, AMP, or Ag(I), the solution was heated to 40 °C for 10 min and then kept at 22 °C for 30 min before recording the fluorescence intensity again.

## Supporting Information

File 1 Results of additional fluorescence spectroscopy assays, CD spectra, additional melting curves, results of additional assays with immobilized ATP, oligonucleotide sequences, MALDI–TOF spectra of the oligonucleotides and HPLC traces to confirm the oligonucleotide purity.
