# Supplementary material for: Incorporation of a metal-mediated base pair into an ATP aptamer – using silver(I) ions to modulate aptamer function
Source: Beilstein J Org Chem. 2020 Nov 25;16:2870–9. doi: 10.3762/bjoc.16.236 (PMC7705865; doi:10.3762/bjoc.16.236)
Supplement: File 1 — Results of additional fluorescence spectroscopy assays, CD spectra, additional melting curves, results of additional assays with immobilized ATP, oligonucleotide sequences, MALDI–TOF spectra of the oligonucleotides and HPLC traces to confirm the oligonucleotide purity. [file Beilstein_J_Org_Chem-16-2870-s001.pdf]

BEILSTEIN JOURNAL OF ORGANIC CHEMISTRY

Supporting Information

for

Incorporation of a metal-mediated base pair into an ATP aptamer – using silver(I) ions to modulate aptamer function

Marius H. Heddinga and Jens Müller

Beilstein J. Org. Chem. **2020,** *16,* 2870–2879. doi:10.3762/bjoc.16.236

Results of additional fluorescence spectroscopy assays, CD spectra, additional melting curves, results of additional assays with immobilized ATP, oligonucleotide sequences, MALDI–TOF spectra of the oligonucleotides and HPLC traces to confirm the oligonucleotide purity

License and Terms: This is a supporting information file under the terms of the Creative Commons Attribution License (https://creativecommons.org/licenses/by/4.0). Please note that the reuse, redistribution and reproduction in particular requires that the authors and source are credited.
The license is subject to the *Beilstein Journal of Organic Chemistry* terms and conditions: (https://www.beilstein-journals.org/bjoc)

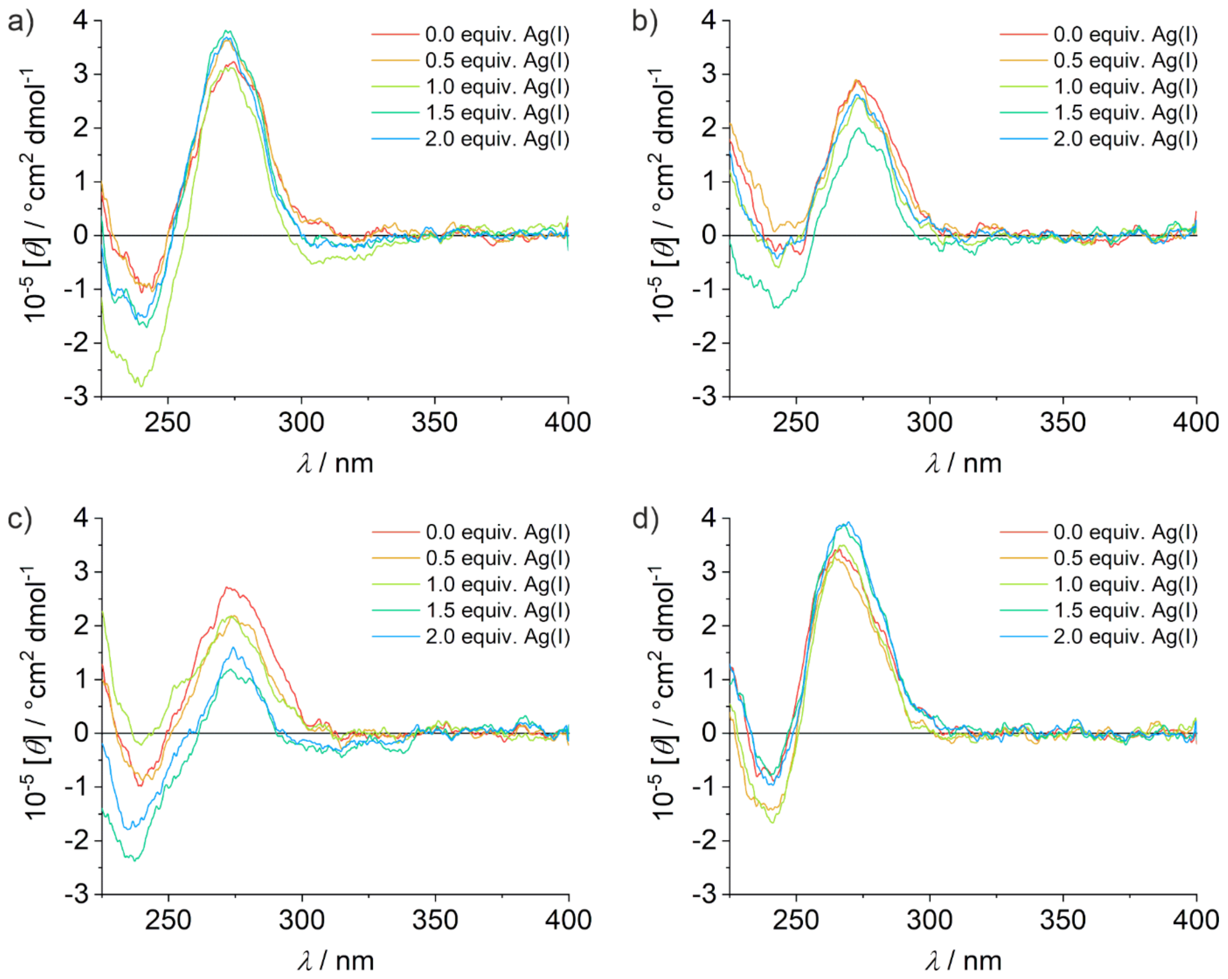

Figure S1: CD spectra of a) **1af**, b) **1bf**, c) **1cf**, and d) **1df** in the absence (red line) and presence of increasing amounts of Ag(I). Conditions: 1 μM oligonucleotide, 50 mM $NaClO_4$, 5 mM MOPS buffer (pH 6.8).

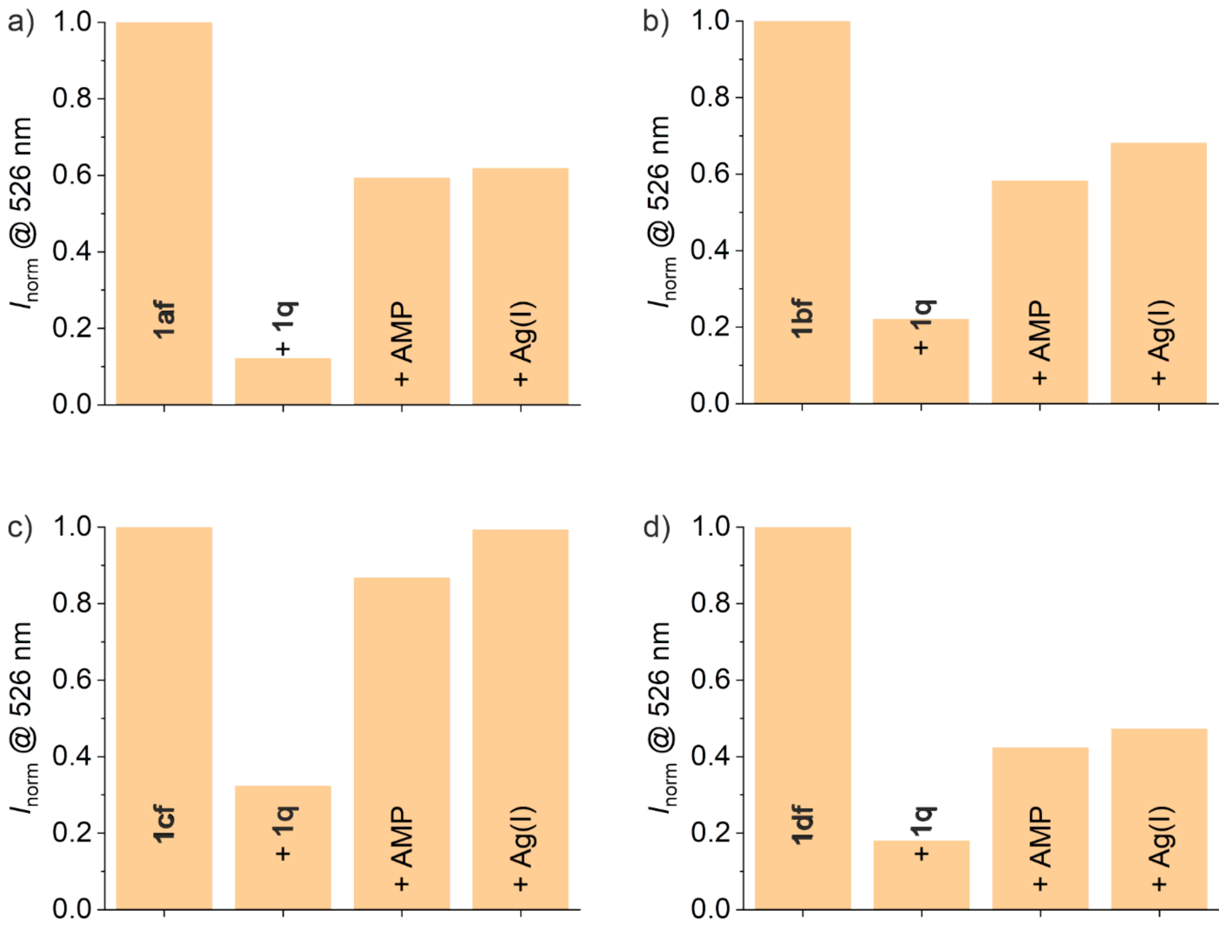

Figure S2: Normalized fluorescence intensity at 526 nm (λ_{exc} = 490 nm) of the aptamer derivatives a) **1af**, b) **1bf**, c) **1cf**, and d) **1df** prior to and after the successive addition of quencher-labeled oligonucleotide **1q**, 1 mM AMP, and 1 equiv of Ag(I). After each addition and before the respective measurement, the solution was heated to 40 °C for 10 min and then kept at 22 °C for 30 min (conditions: 40 nM fluorophore-labeled DNA; 80 nM quencher-labeled DNA; 300 mM $NaClO_4$; 5 mM $Mg(ClO_4)_2$; 20 mM MOPS buffer (pH 8.1)).

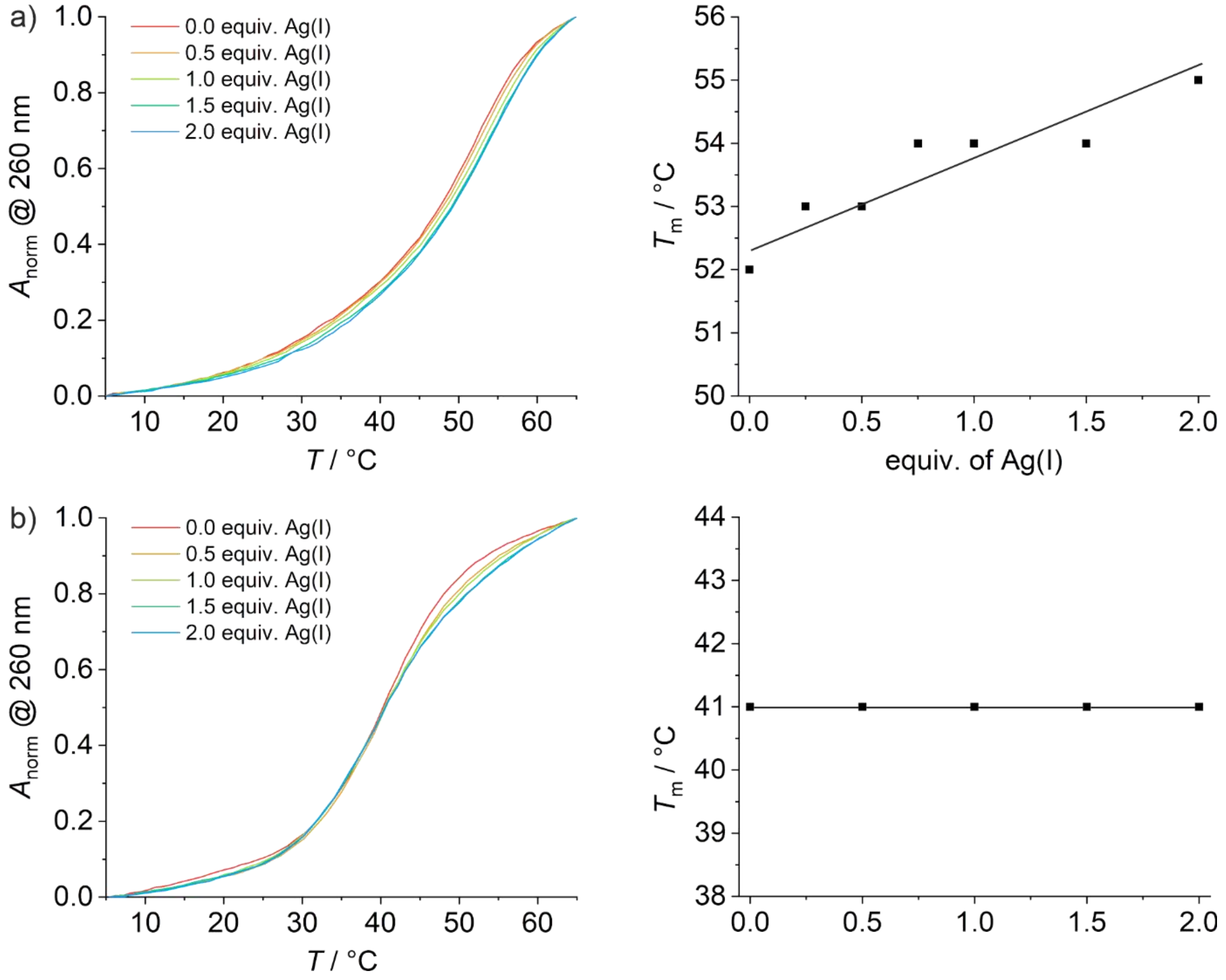

Figure S3: Temperature-dependent normalized UV absorbance at 260 nm and melting temperatures T_m for the duplexes formed from oligonucleotides a) **1af** and **1q** as well as b) **1a**, **2f**, and **2q**. Conditions: 1 μM oligonucleotide, 50 mM $NaClO_4$, 5 mM MOPS buffer (pH 6.8).

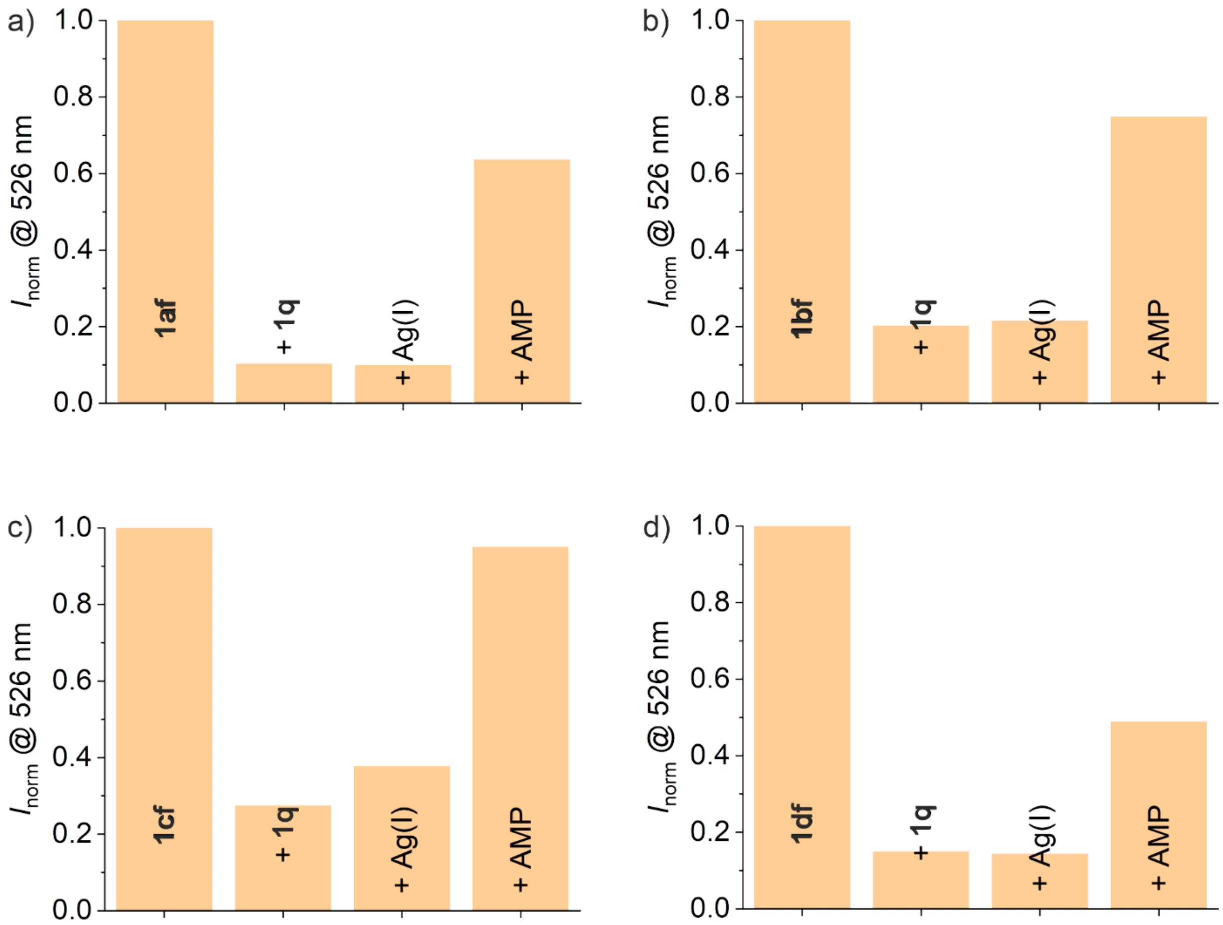

Figure S4: Normalized fluorescence intensity at 526 nm (λ_{exc} = 490 nm) of the aptamer derivatives a) **1af**, b) **1bf**, c) **1cf**, and d) **1df** prior to and after the successive addition of quencher-labeled oligonucleotide **1q**, 1 equiv of Ag(I), and 10 mM AMP. After each addition and before the respective measurement, the solution was heated to 40 °C for 10 min and then kept at 22 °C for 30 min (conditions: 40 nM fluorophore-labelled DNA; 80 nM quencher-labelled DNA; 300 mM $NaClO_4$; 5 mM $Mg(ClO_4)_2$; 20 mM MOPS buffer (pH 8.1)).

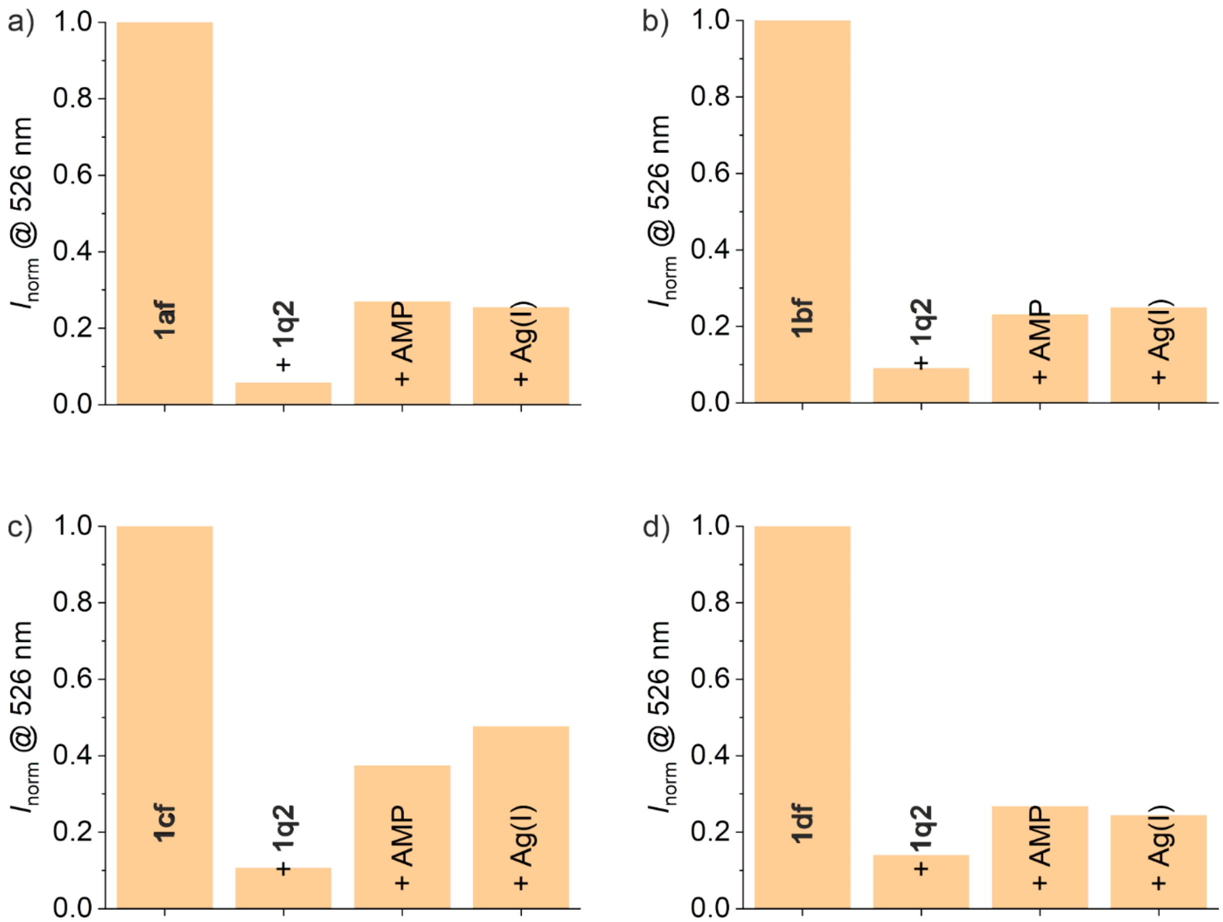

Figure S5: Normalized fluorescence intensity at 526 nm (λ_{exc} = 490 nm) of the aptamers a) **1af**, b) **1bf**, c) **1cf**, and d) **1df** prior to and after the successive addition of the extended quencher-labeled oligonucleotide **1q2**, 1 mM AMP, and 1 equiv of Ag(I). After every addition and before the respective measurement, the solution was heated to 50 °C for 10 min and then kept at 22 °C for 30 min (conditions: 40 nM fluorophore-labelled DNA; 80 nM quencher-labelled DNA; 300 mM $NaClO_4$; 5 mM $Mg(ClO_4)_2$; 20 mM MOPS buffer (pH 8.1)).

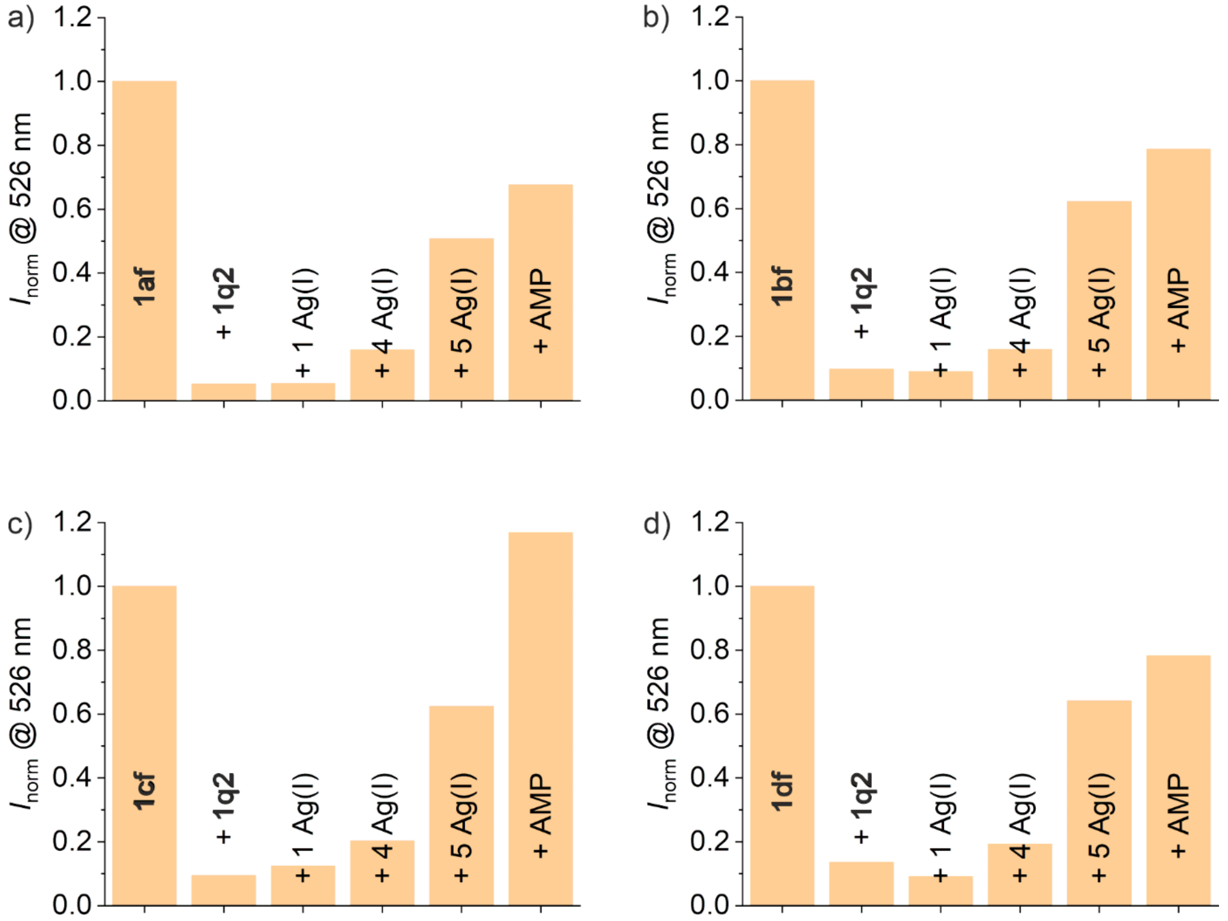

Figure S6: Normalized fluorescence intensity at 526 nm (λ_{exc} = 490 nm) of the aptamers a) **1af**, b) **1bf**, c) **1cf**, and d) **1df** prior to and after the successive addition of the extended quencher oligonucleotide **1q2**, 1 equiv of Ag(I), 4 more equiv of Ag(I), 5 more equiv of Ag(I), and 1 mM AMP. After every addition and before the respective measurement, the solution was heated to 50 °C for 10 min and then kept at 22 °C for 30 min (conditions: 40 nM fluorophore-labelled DNA; 80 nM quencher-labelled DNA; 300 mM $NaClO_4$; 5 mM $Mg(ClO_4)_2$; 20 mM MOPS buffer (pH 8.1)).

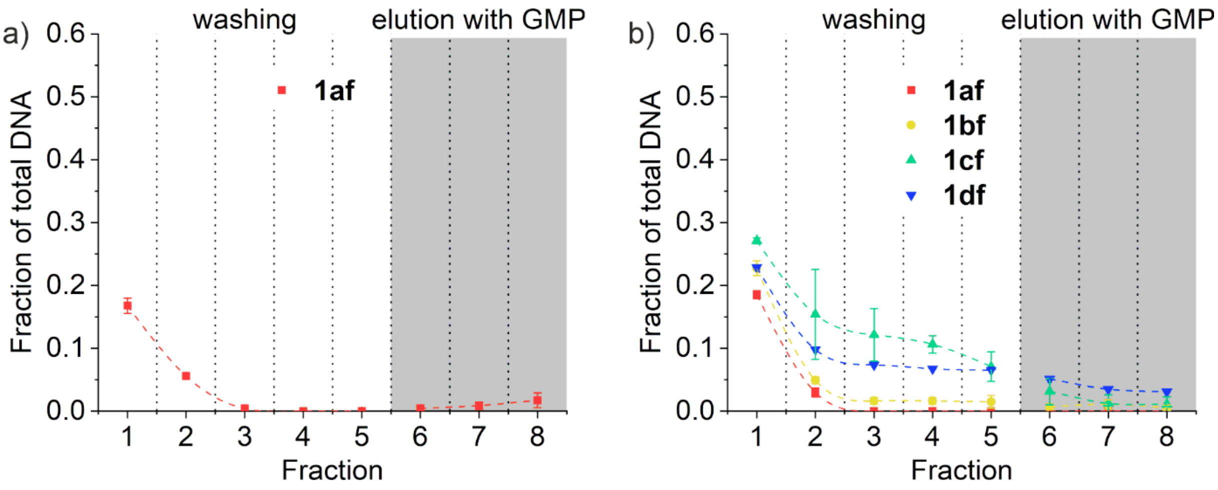

Figure S7: Elution of aptamers from the ATP-agarose gel by means of a GMP solution in a) the absence and b) the presence of 1 equiv of Ag(I): **1a** (red), **1b** (yellow), **1c** (green), and **1d** (blue) (n = 3; for **1d**: n = 2). Conditions: 1 μM oligonucleotide, 300 mM $NaClO_4$, 5 mM $Mg(ClO_4)_2$, 20 mM MOPS buffer (pH 7.4).

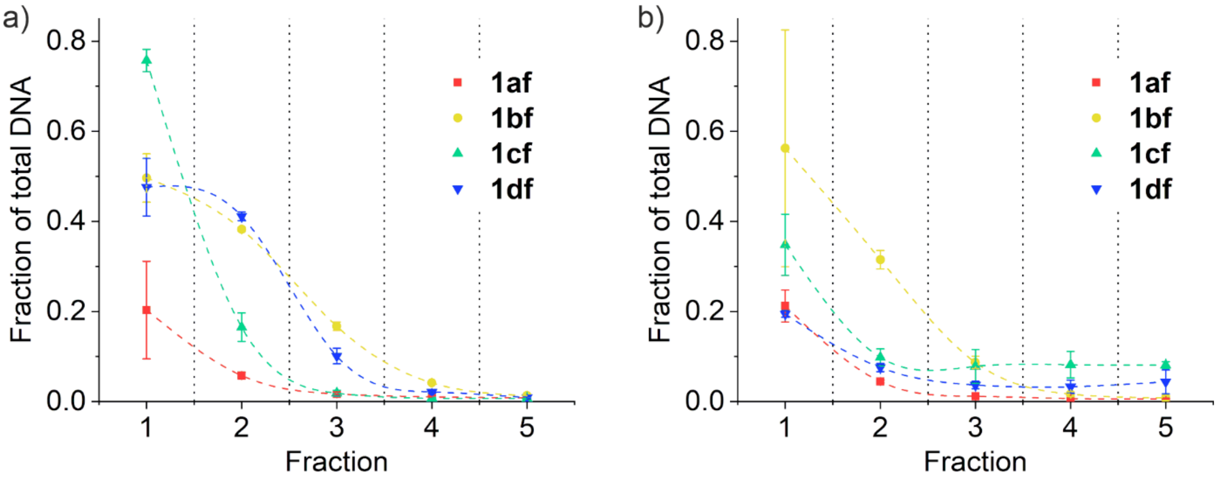

Figure S8: Elution of the unlabeled aptamers from the ATP-agarose gel in a) the absence and b) the presence of 1 equiv of Ag(I): **1a** (red), **1b** (yellow), **1c** (green), and **1d** (blue) (n = 3). Conditions: 1 μM oligonucleotide, 300 mM $NaClO_4$, 5 mM $Mg(ClO_4)_2$, 20 mM MOPS buffer (pH 7.4).

Table S1: Oligonucleotides used in this study and their mass-spectrometric characterization.

	Sequence	Formula	Calcd. mass for $[M+H]^+$	Exp. mass
**1af**	5´-d(**F**-ACC-TGG-GGG-AGT-ATT-GCG-GAG-GAA-GGT)	$C_{294}H_{356}N_{115}O_{168}P_{27}$	9022	9024
**1bf**	5´-d(**F**-AC**Im**-TGG-GGG-AGT-ATT-GCG-GAG-GAA-**Im**GT)	$C_{291}H_{354}N_{111}O_{166}P_{27}$	8896	8898
**1cf**	5´-d(**F**-ACC-**Im**GG-GGG-AGT-ATT-GCG-GAG-GA**Im**-GGT)	$C_{290}H_{353}N_{112}O_{166}P_{27}$	8897	8899
**1df**	5´-d(**F**-ACC-TGG-GGG-A**Im**T-ATT-G**Im**G-GAG-GAA-GGT)	$C_{291}H_{354}N_{111}O_{166}P_{27}$	8896	8899
**1q**	5´-d(ACT-CCC-CCA-GGT-**Q**)	$C_{135}H_{176}N_{46}O_{76}P_{12}$	4052 *	4053
**1q2**	5´-d(ATA-CTC-CCC-CAG-GT-**Q**)	$C_{155}H_{201}N_{53}O_{88}P_{14}$	4669 *	4673
**1a**	5´-d(ACC-TGG-GGG-AGT-ATT-GCG-GAG-GAA-GGT)	$C_{267}H_{331}N_{114}O_{159}P_{26}$	8483	8484
**1b**	5´-d(AC**Im**-TGG-GGG-AGT-ATT-GCG-GAG-GAA-**Im**GT)	$C_{264}H_{329}N_{110}O_{157}P_{26}$	8357	8357
**1c**	5´-d(ACC-**Im**GG-GGG-AGT-ATT-GCG-GAG-GA**Im**-GGT)	$C_{263}H_{328}N_{111}O_{157}P_{26}$	8358	8362
**1d**	5´-d(ACC-TGG-GGG-A**Im**T-ATT-G**Im**G-GAG-GAA-GGT)	$C_{264}H_{329}N_{110}O_{157}P_{26}$	8357	8362
**2f**	5´-d(**F**-ATA-CTC-CCC-CA)	$C_{131}H_{161}N_{38}O_{72}P_{11}$	3760	3758
**2q**	5´-d(TCC-TCC-GCA-**Q**)	$C_{106}H_{140}N_{33}O_{59}P_9$	3099	3097

*: Calculated mass for $[M + Na]^+$ rather than $[M + H]^+$

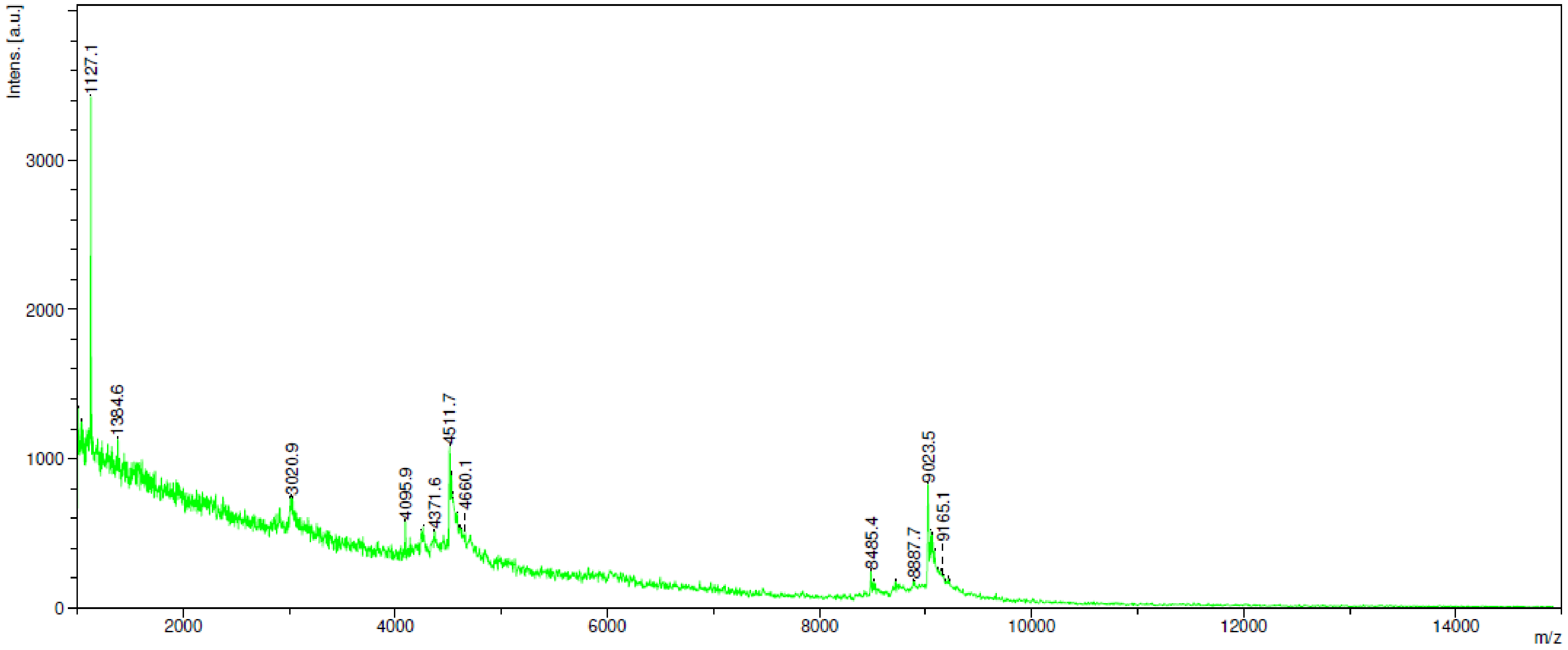

Figure S9: MALDI-TOF mass spectrum of oligonucleotide **1af** ($C_{294}H_{356}N_{115}O_{168}P_{27}$, calcd. for $[M + H]^+$: 9022 Da, found: 9024 Da).

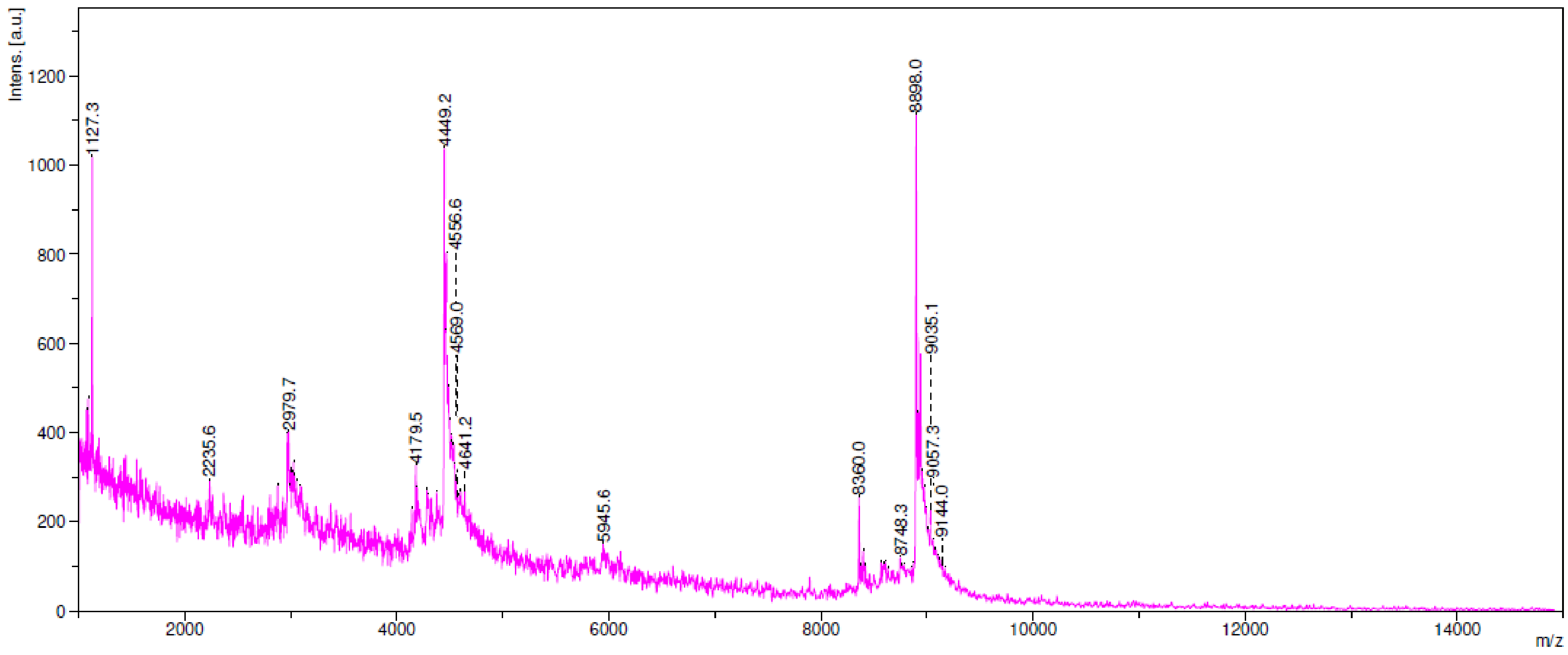

Figure S10: MALDI-TOF mass spectrum of oligonucleotide **1bf** ($C_{291}H_{354}N_{111}O_{166}P_{27}$, calcd. for $[M + H]^+$: 8896 Da, found: 8898 Da).

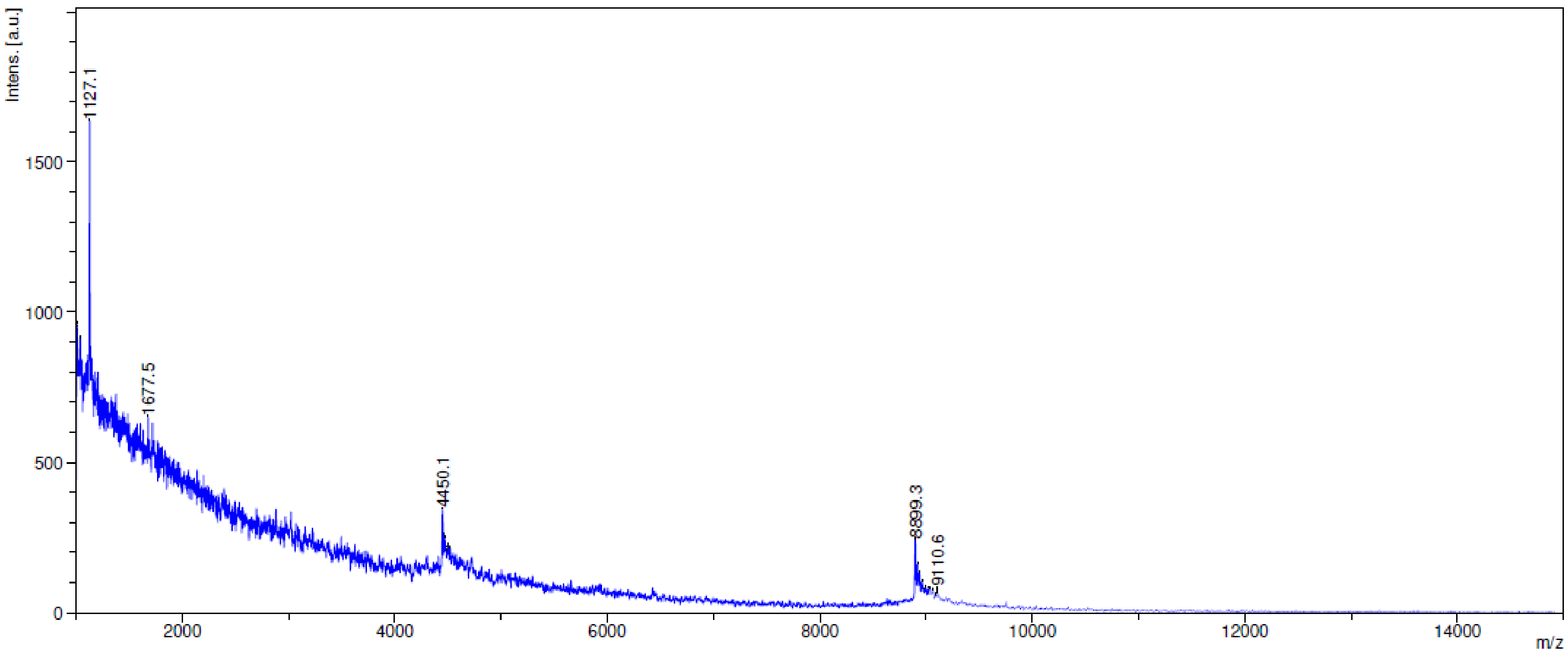

Figure S11: MALDI-TOF mass spectrum of oligonucleotide **1cf** ($C_{290}H_{353}N_{112}O_{166}P_{27}$, calcd. for $[M + H]^+$: 8897 Da, found: 8899 Da).

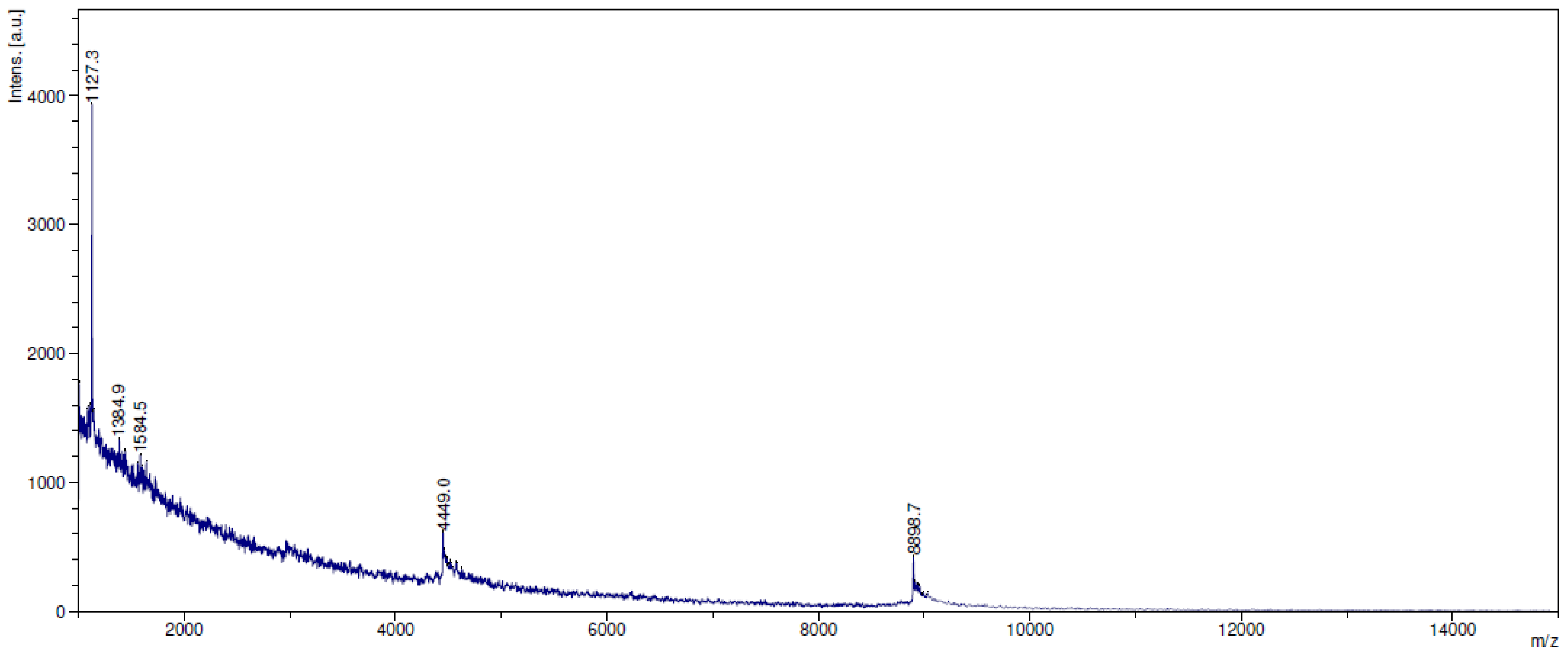

Figure S12: MALDI-TOF mass spectrum of oligonucleotide **1df** ($C_{291}H_{354}N_{111}O_{166}P_{27}$, calcd. for $[M + H]^+$: 8896 Da, found: 8899 Da).

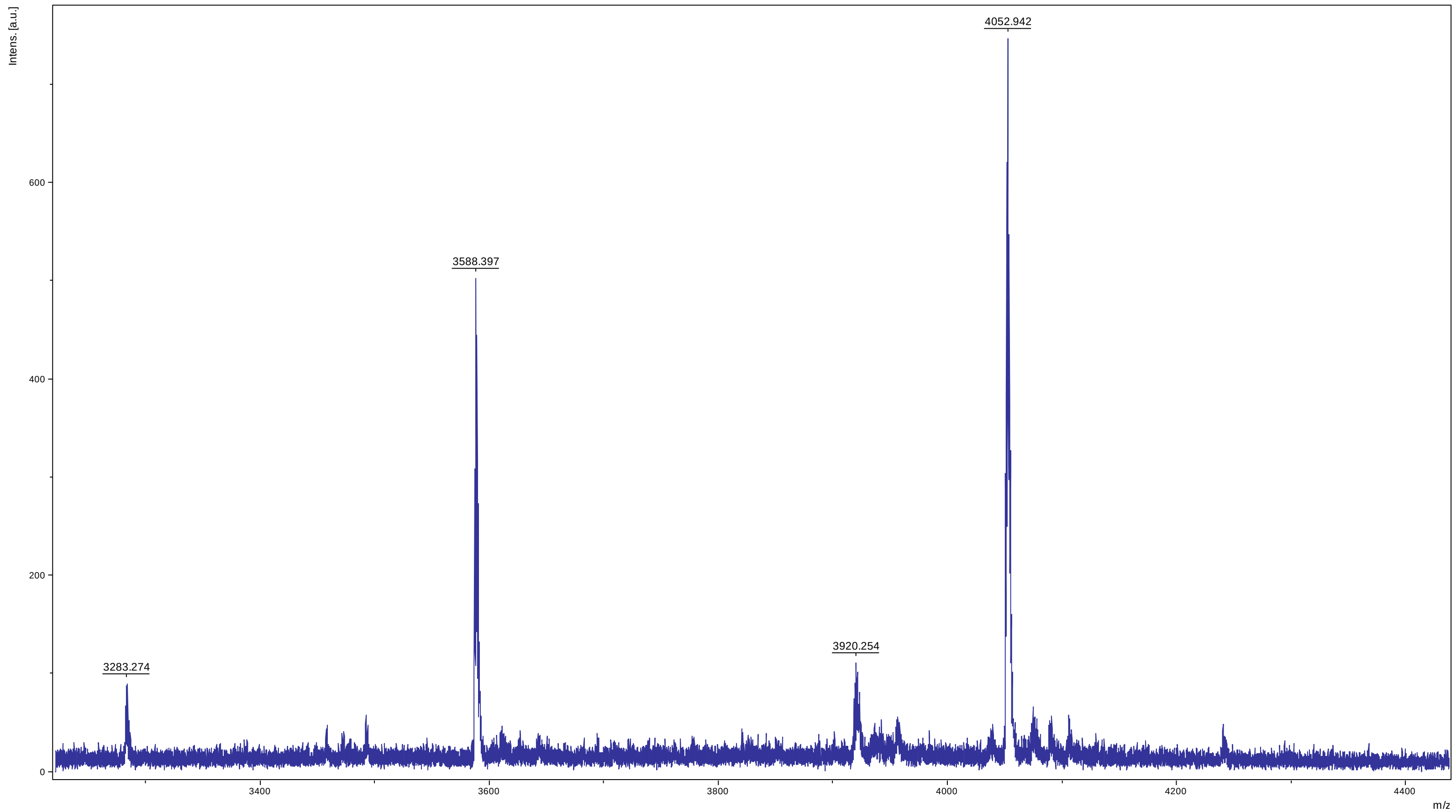

Figure S13: MALDI-TOF mass spectrum of oligonucleotide **1q** ($C_{135}H_{176}N_{46}O_{76}P_{12}$, calcd. for $[M + Na]^+$: 4052 Da, found: 4053 Da). The peak at *m*/*z* = 3588 represents the oligonucleotide lacking its quencher moiety ($C_{114}H_{148}N_{42}O_{70}P_{11}$, calcd. for $[M + Na]^+$: 3589 Da, found: 3588 Da). As the quencher is attached at the 3' end of the oligonucleotide, its loss is likely to have occurred during the mass-spectrometric characterization of the product.

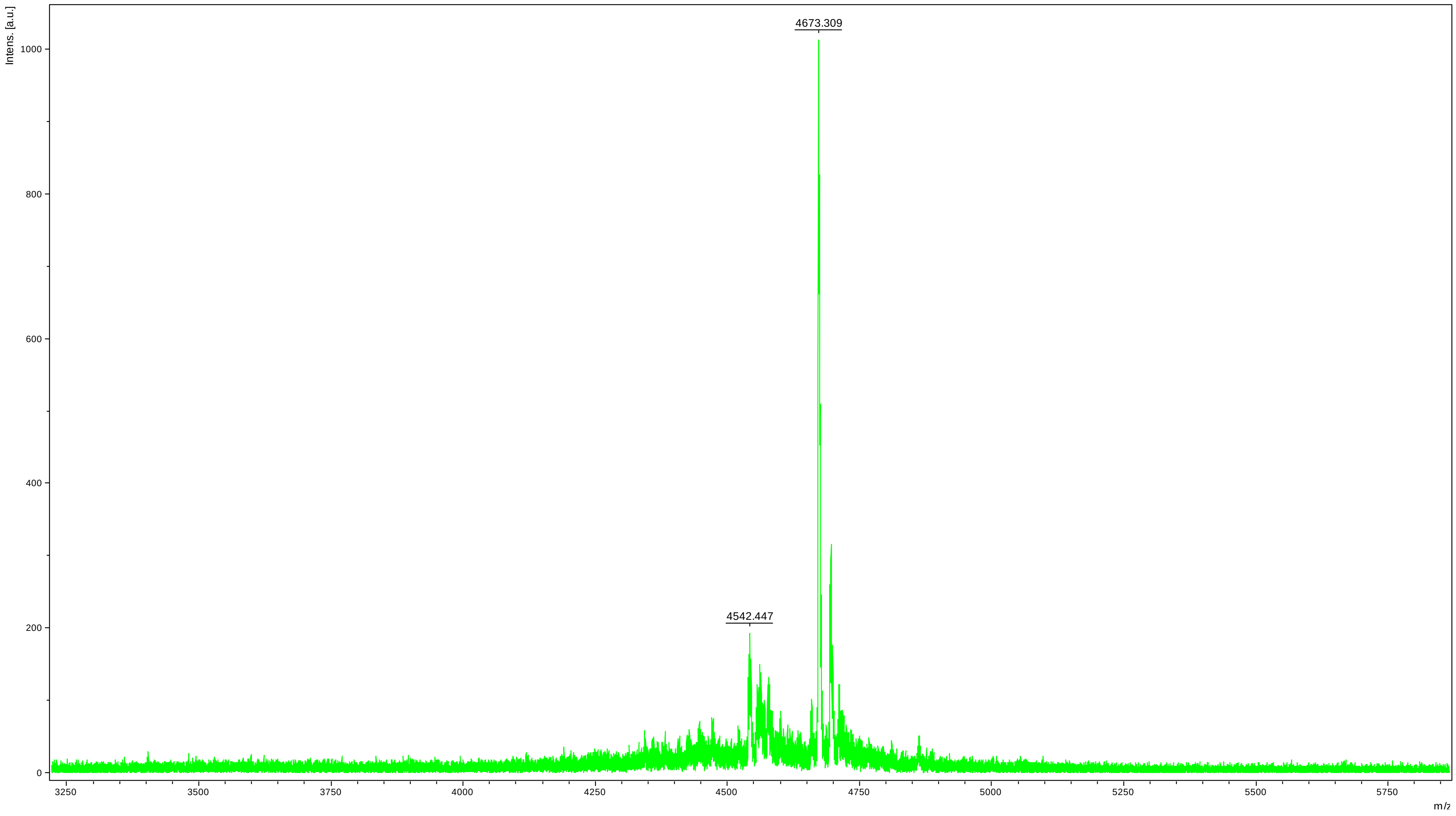

Figure S14: MALDI-TOF mass spectrum of oligonucleotide **1q2** ($C_{155}H_{201}N_{53}O_{88}P_{14}$, calcd. for $[M + Na]^+$: 4669 Da, found: 4673 Da).

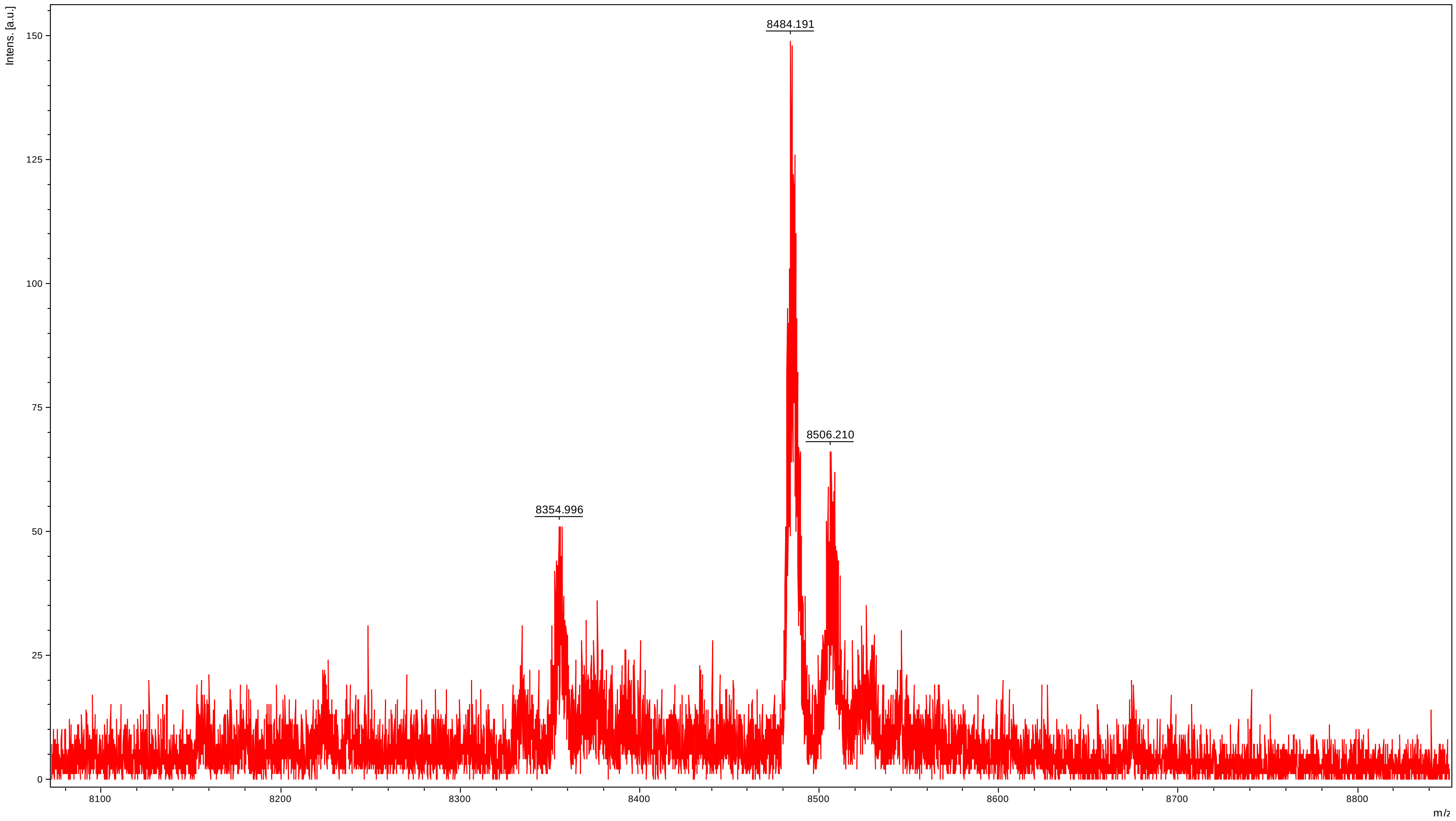

Figure S15: MALDI-TOF mass spectrum of oligonucleotide **1a** ($C_{267}H_{331}N_{114}O_{159}P_{26}$, calcd. for $[M + H]^+$: 8483 Da, found: 8484 Da).

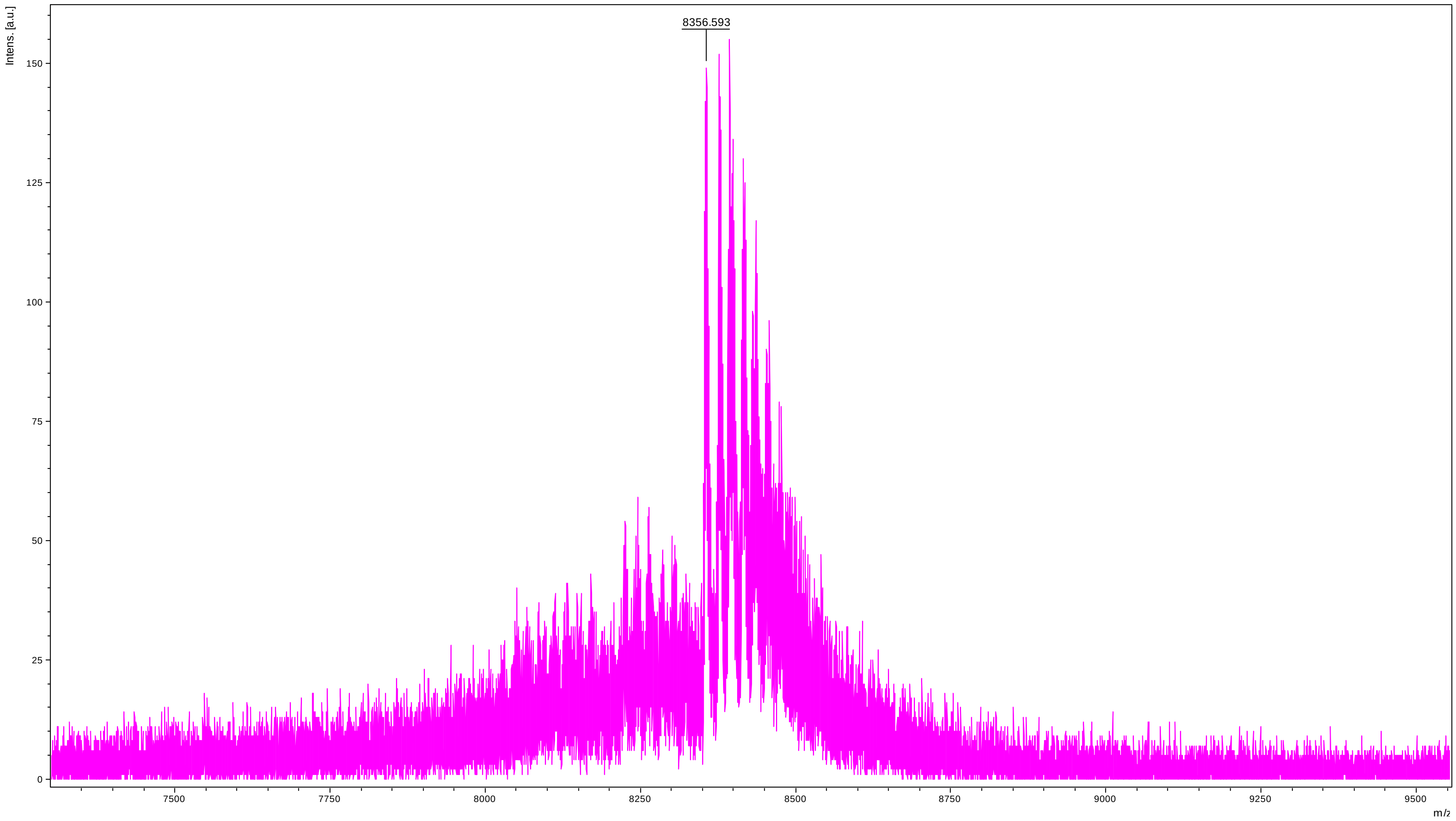

Figure S16: MALDI-TOF mass spectrum of oligonucleotide **1b** ($C_{264}H_{329}N_{110}O_{157}P_{26}$, calcd. for $[M + H]^+$: 8357 Da, found: 8357 Da).

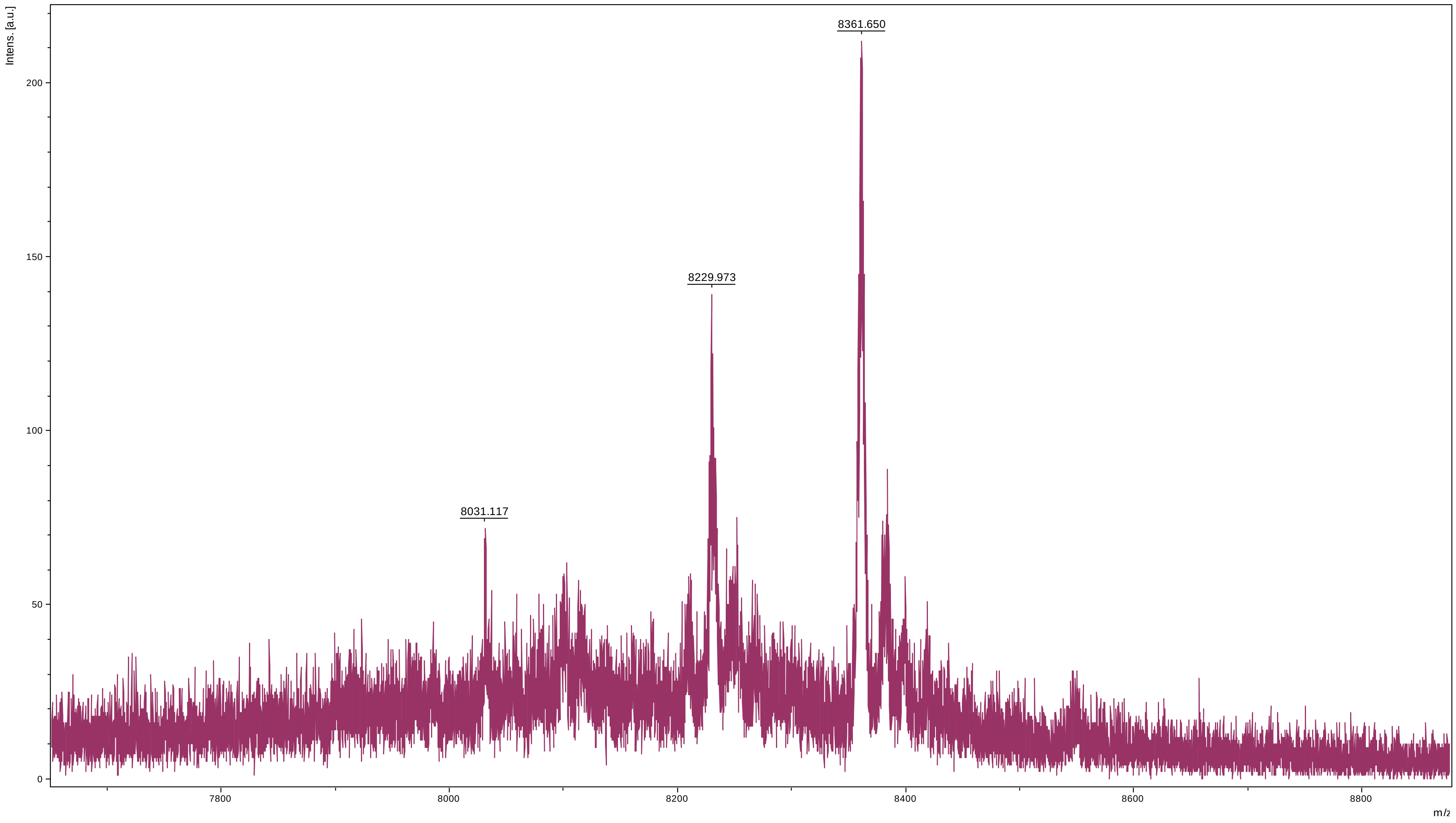

Figure S17: MALDI-TOF mass spectrum of oligonucleotide **1c** ($C_{263}H_{328}N_{111}O_{157}P_{26}$, calcd. for $[M + H]^+$: 8358 Da, found: 8362 Da).

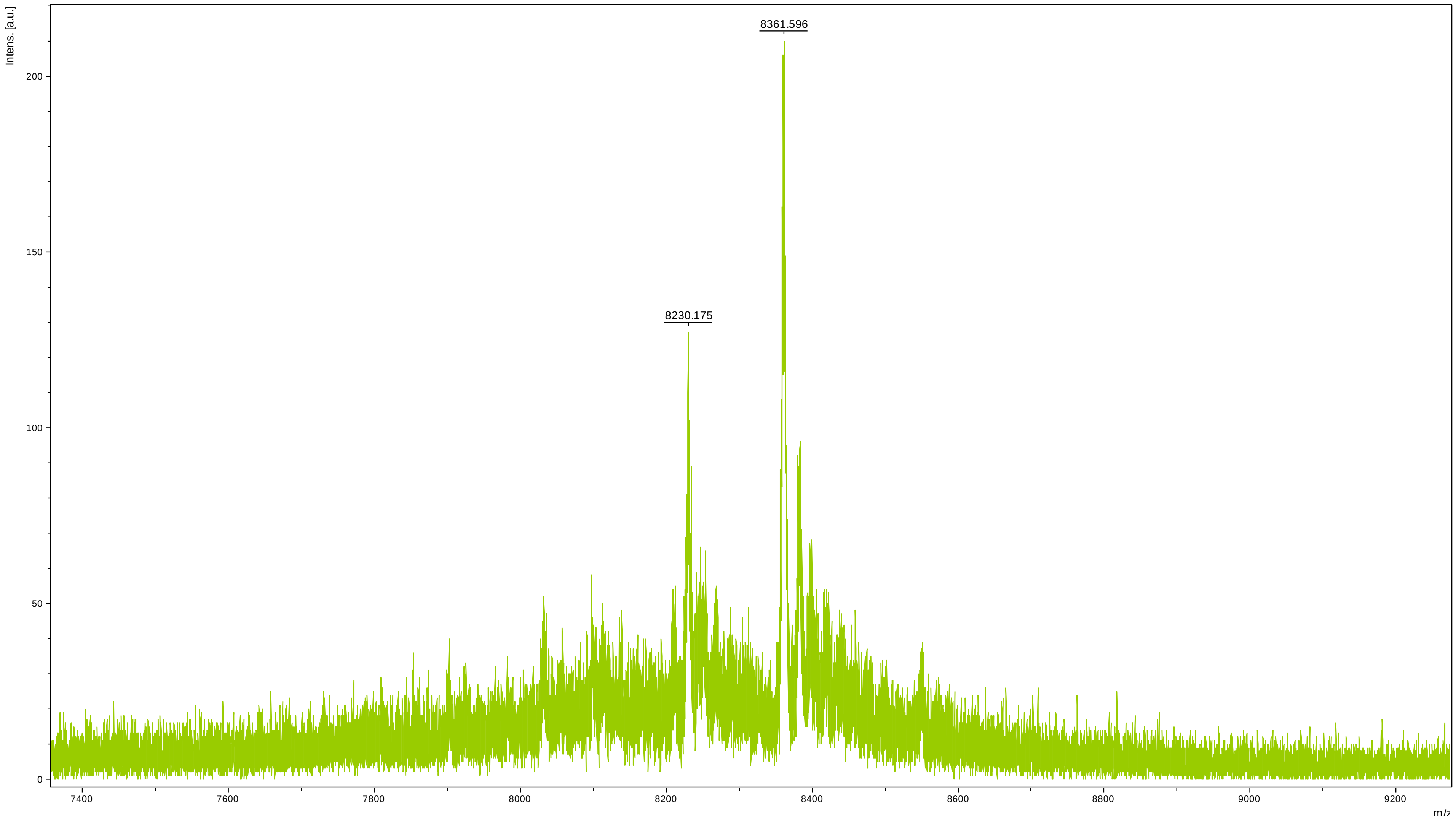

Figure S18: MALDI-TOF mass spectrum of oligonucleotide **1d** ($C_{264}H_{329}N_{110}O_{157}P_{26}$, calcd. for $[M + H]^+$: 8357 Da, found: 8362 Da).

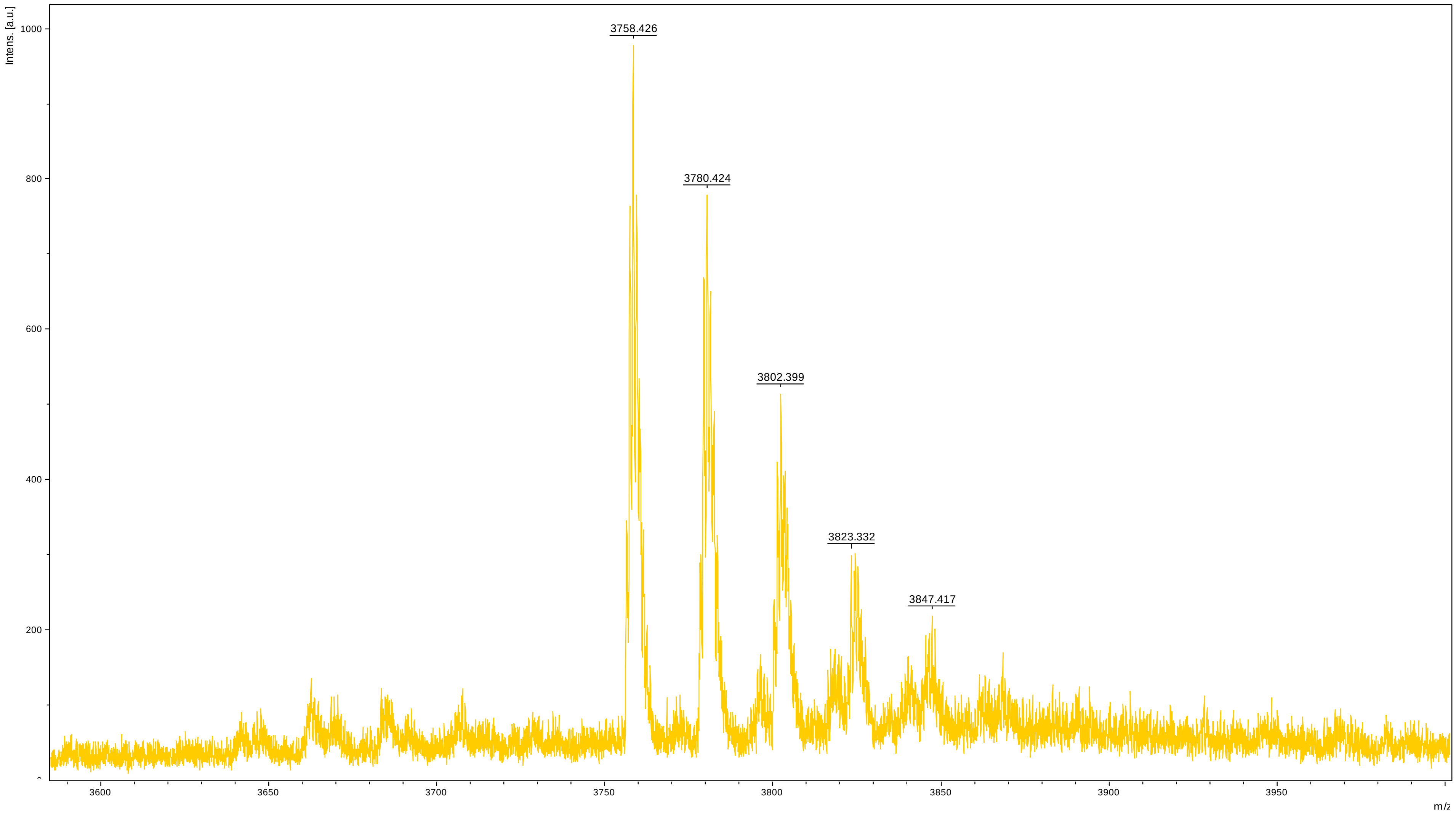

Figure S19: MALDI-TOF mass spectrum of oligonucleotide **2f** ($C_{131}H_{161}N_{38}O_{72}P_{11}$, calcd. for $[M + H]^+$: 3760 Da, found: 3758 Da).

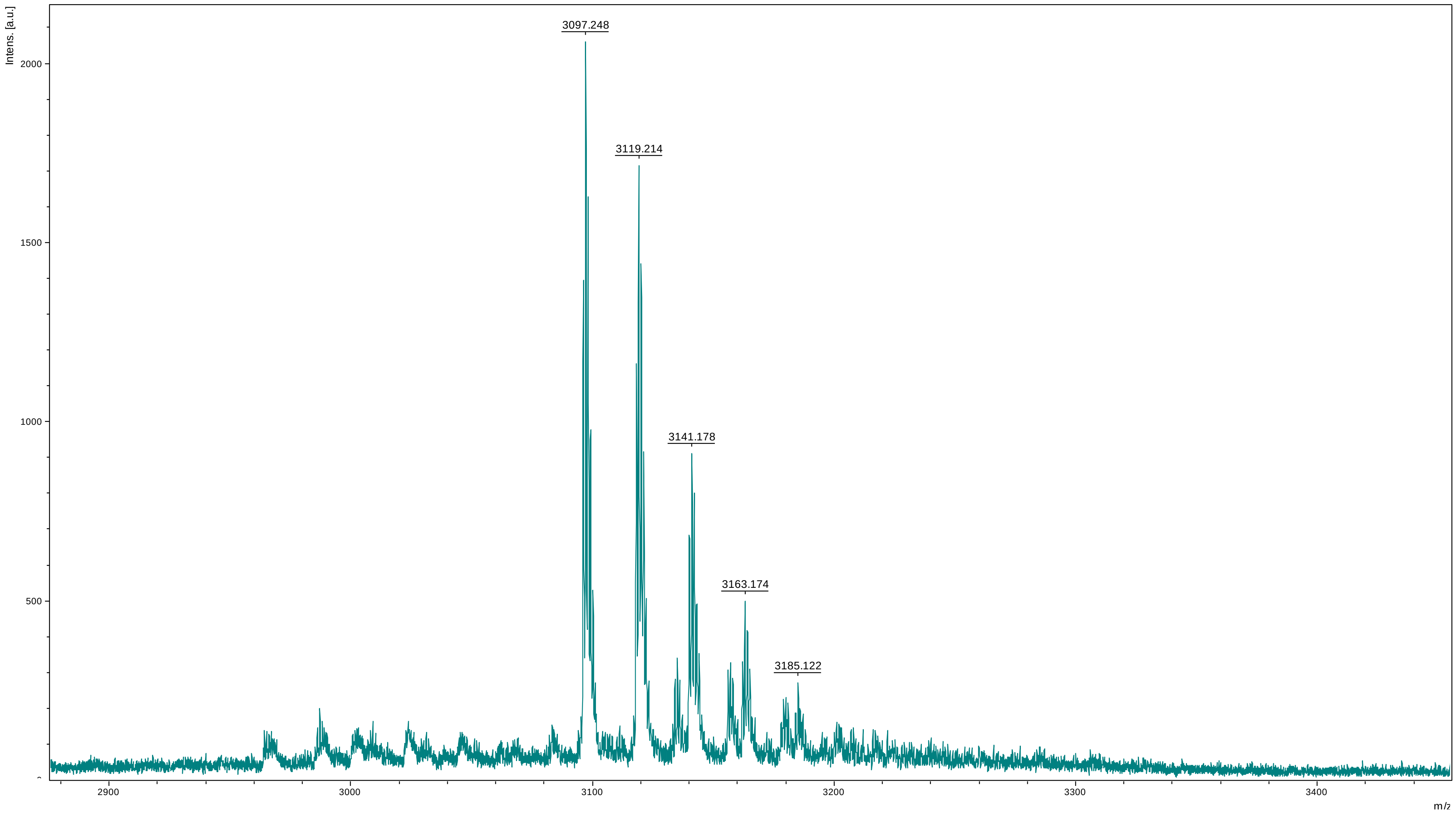

Figure S20: MALDI-TOF mass spectrum of oligonucleotide **2q** ($C_{106}H_{140}N_{33}O_{59}P_9$, calcd. for $[M + H]^+$: 3099 Da, found: 3097 Da).

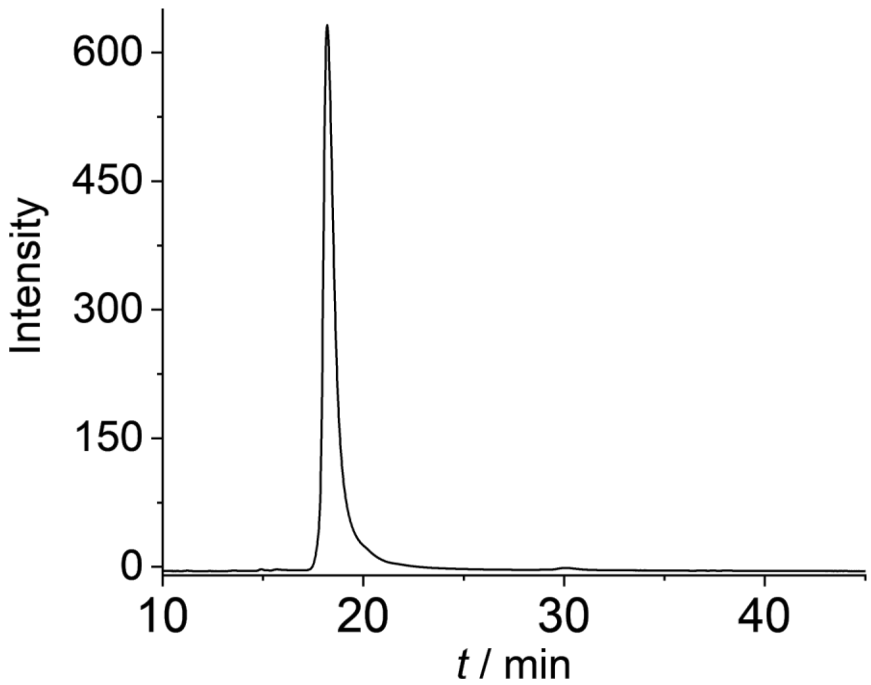

Figure S21: HPLC chromatogram of oligonucleotide **1a** (absorbance at 260 nm).

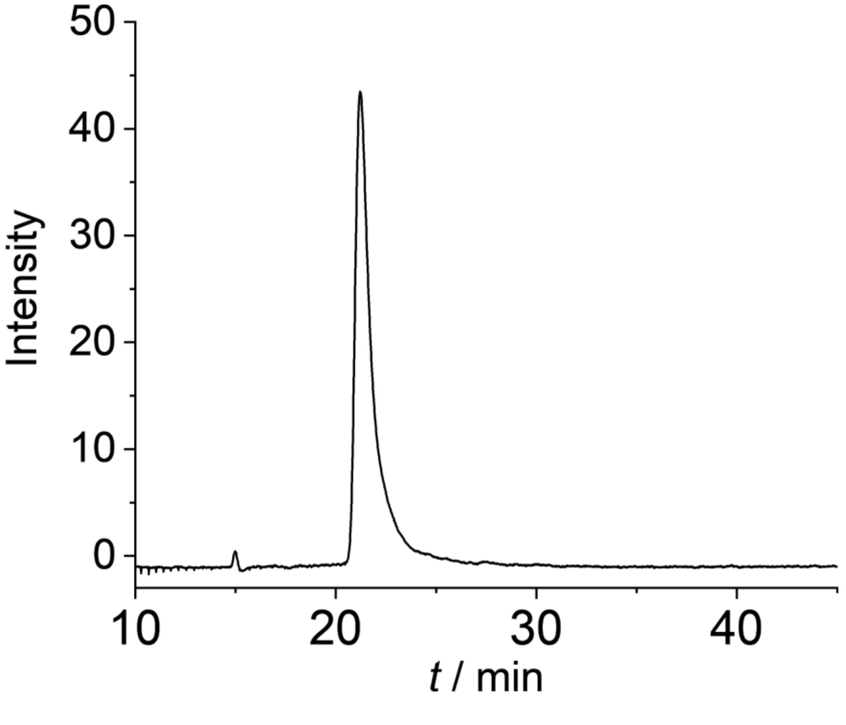

Figure S22: HPLC chromatogram of oligonucleotide **1af** (absorbance at 488 nm).

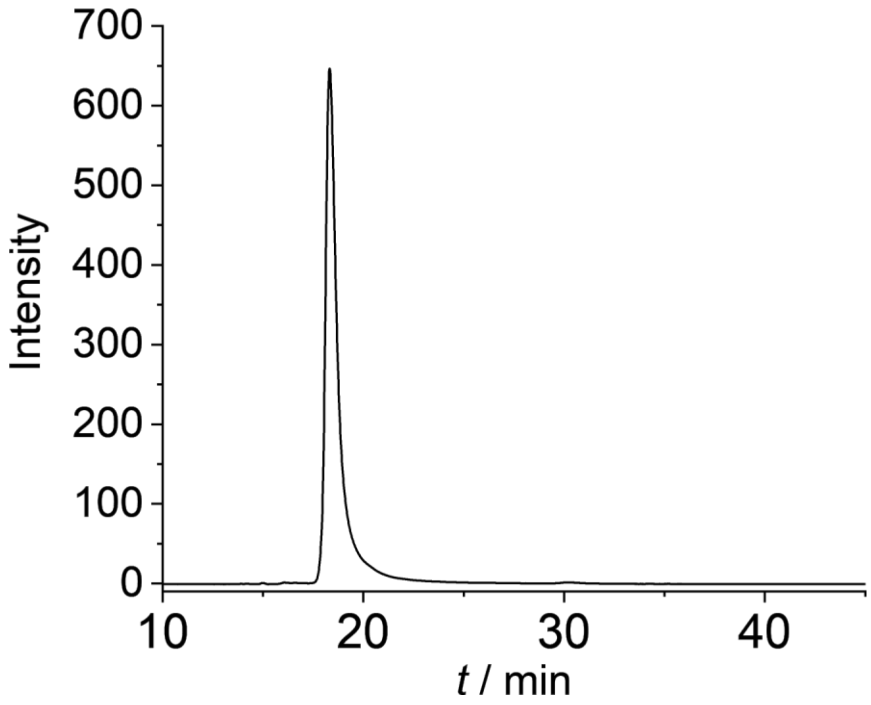

Figure S23: HPLC chromatogram of oligonucleotide **1b** (absorbance at 260 nm).

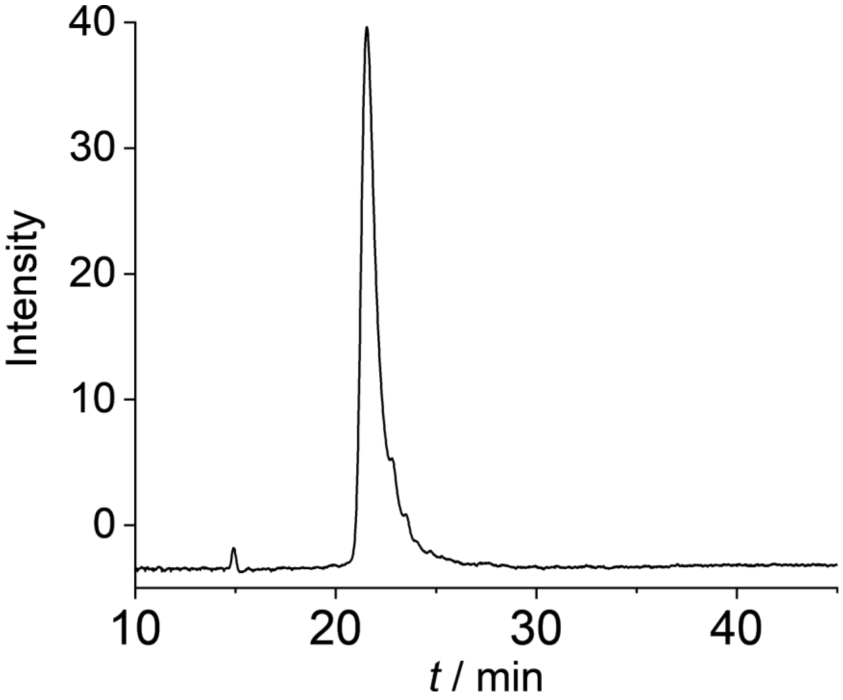

Figure S24: HPLC chromatogram of oligonucleotide **1bf** (absorbance at 488 nm).

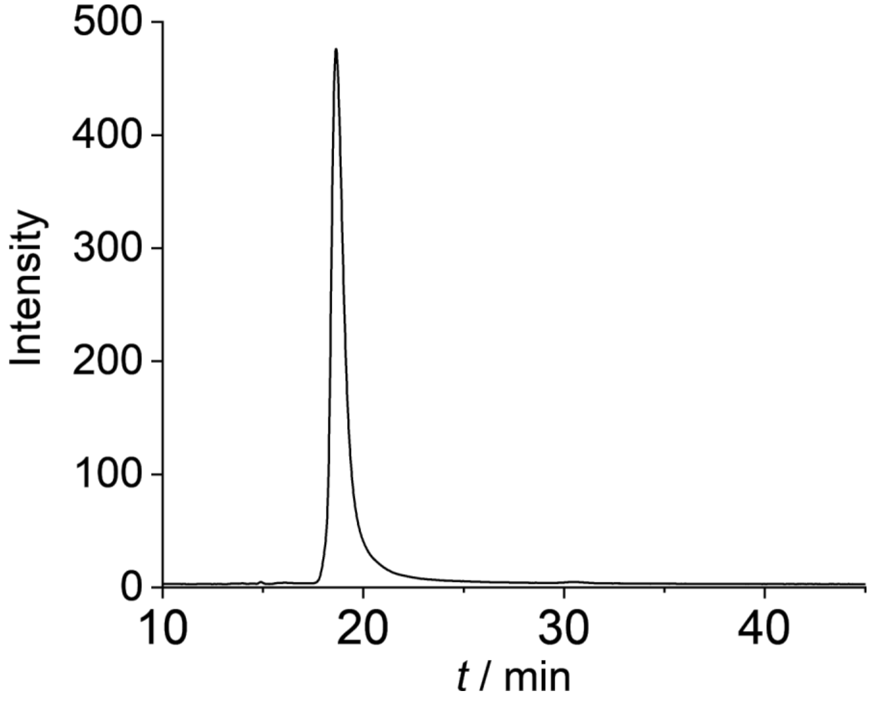

Figure S25: HPLC chromatogram of oligonucleotide **1c** (absorbance at 260 nm).

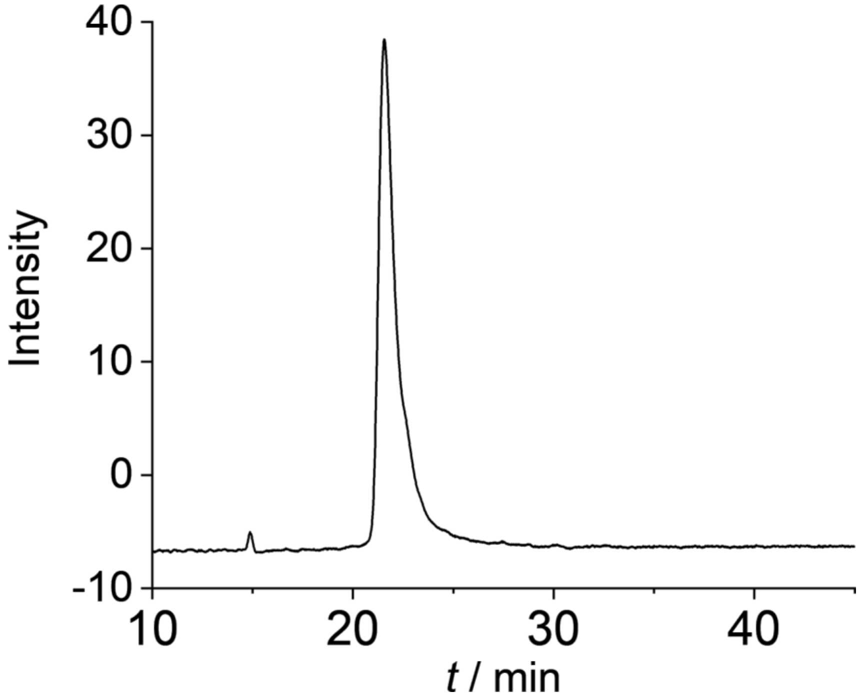

Figure S26: HPLC chromatogram of oligonucleotide **1cf** (absorbance at 488 nm).

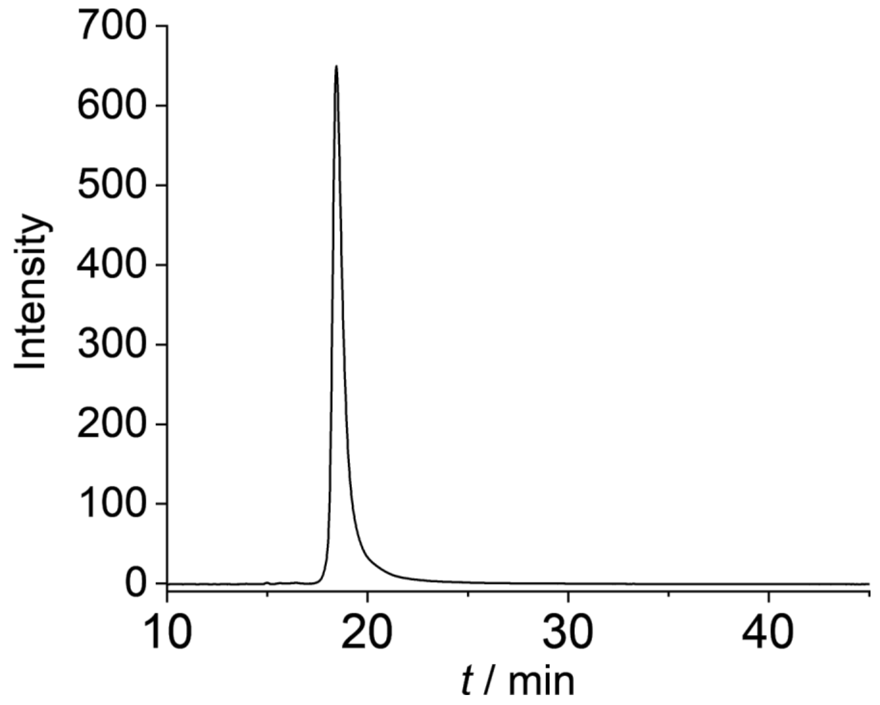

Figure S27: HPLC chromatogram of oligonucleotide **1d** (absorbance at 260 nm).

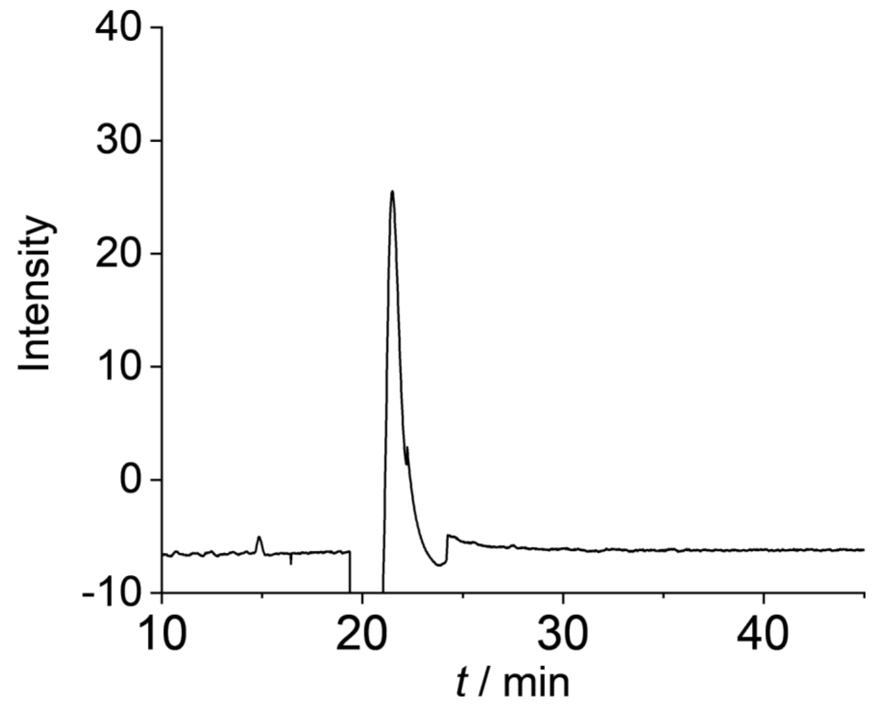

Figure S28: HPLC chromatogram of oligonucleotide **1df** (absorbance at 488 nm).

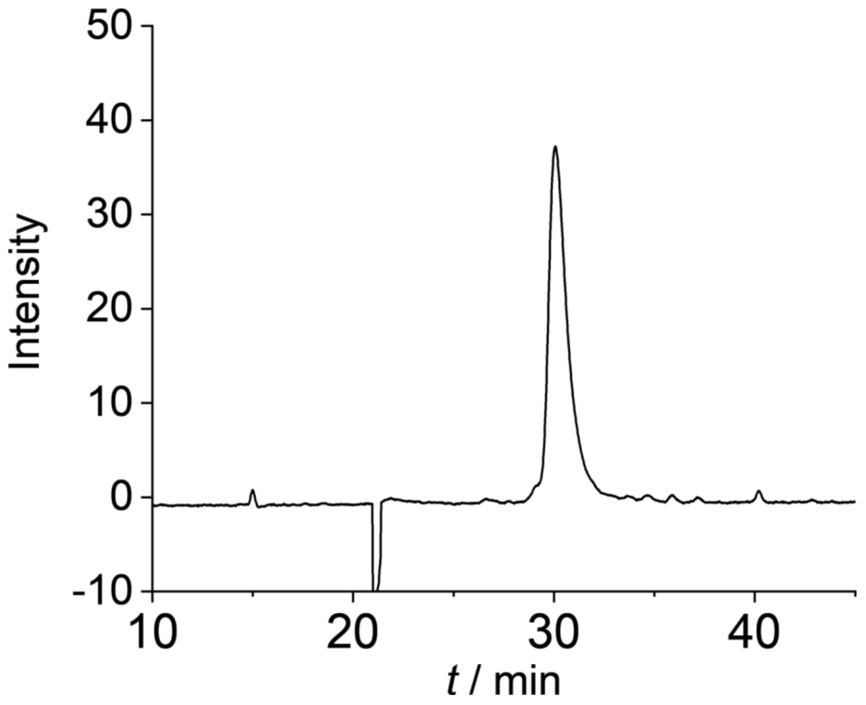

Figure S29: HPLC chromatogram of oligonucleotide **1q** (absorbance at 472 nm).

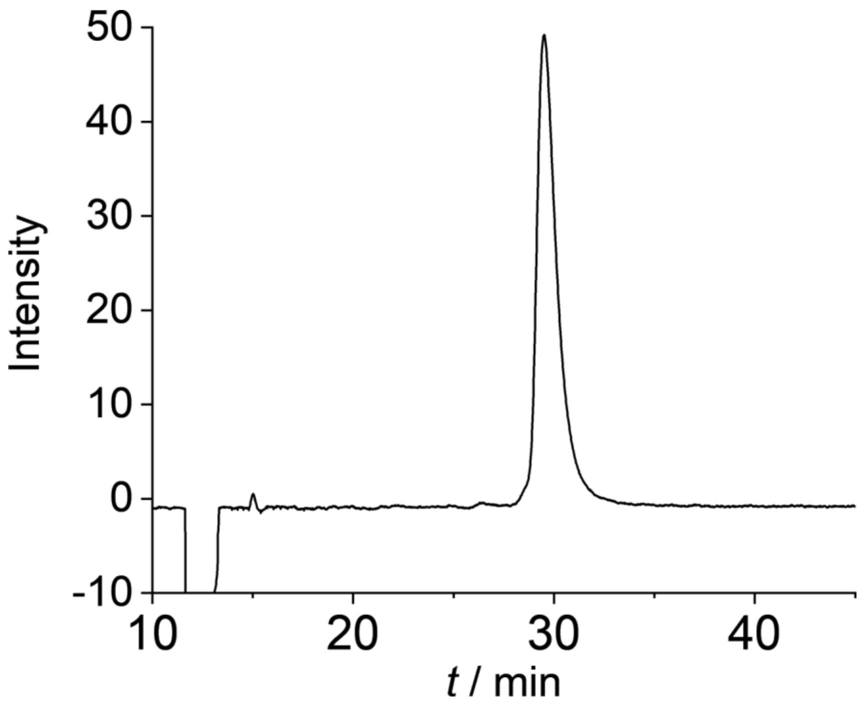

Figure S30: HPLC chromatogram of oligonucleotide **1q2** (absorbance at 472 nm).

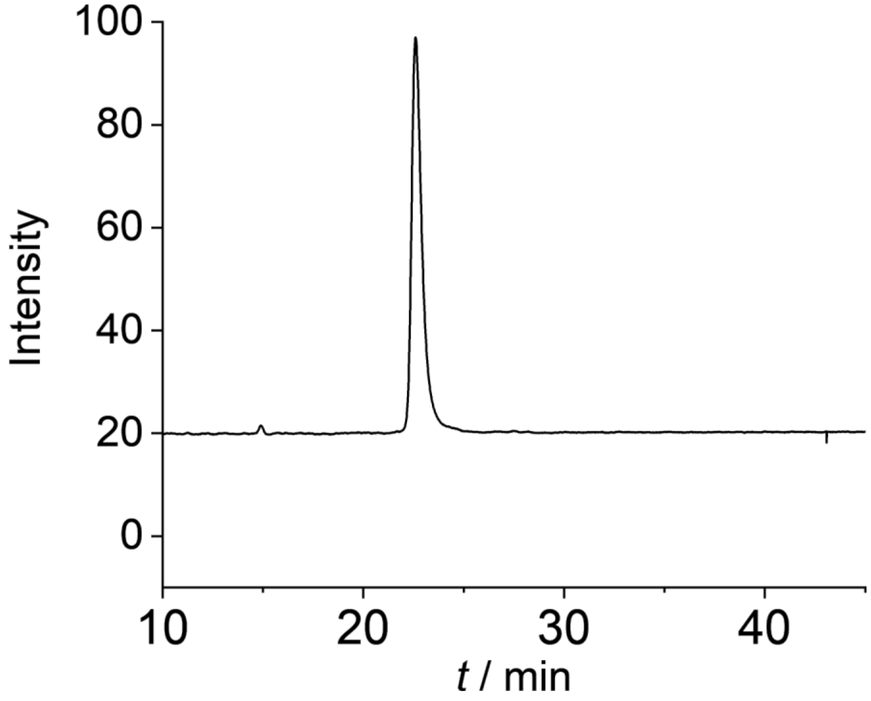

Figure S31: HPLC chromatogram of oligonucleotide **2f** (absorbance at 488 nm).

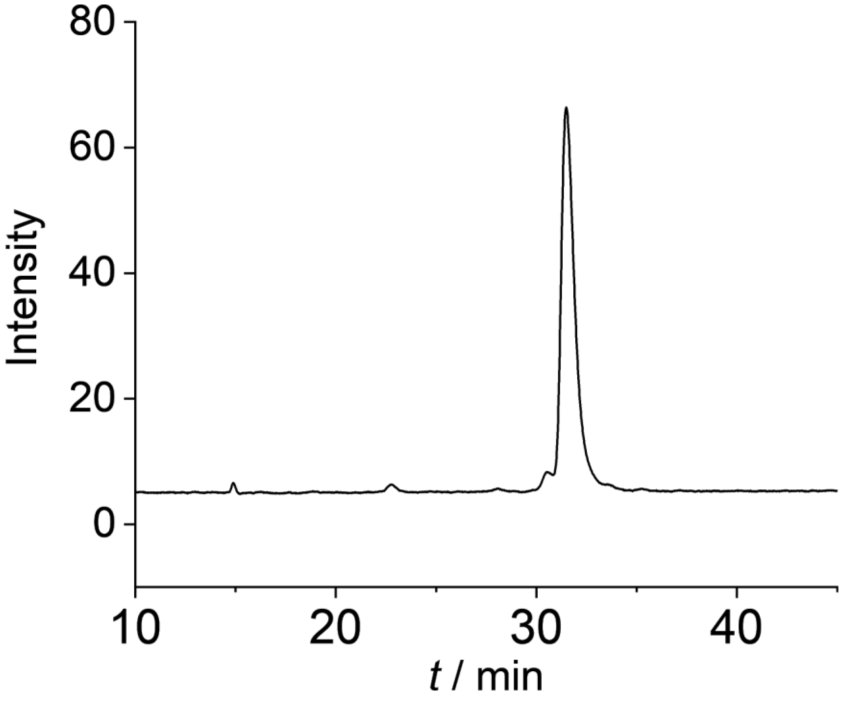

Figure S32: HPLC chromatogram of oligonucleotide **2q** (absorbance at 472 nm).
